# A Novel Sprayable Fibrinogen/Glycosaminoglycans/Collagen‐Based Bioink for Skin Wound Healing Applied by a Handheld Dual‐Head Airbrush

**DOI:** 10.1002/adhm.202500702

**Published:** 2025-07-25

**Authors:** Paula Pleguezuelos‐Beltrán, Daniel Nieto‐García, Carlos Chocarro‐Wrona, Juan de Vicente, Patricia Gálvez‐Martín, José Manuel Entrena, Elena López‐Ruiz, Juan Antonio Marchal

**Affiliations:** ^1^ Biopathology and Regenerative Medicine Institute (IBIMER) Center for Biomedical Research (CIBM) University of Granada Granada 18016 Spain; ^2^ Instituto de Investigación Biosanitaria ibs.GRANADA Granada 18012 Spain; ^3^ Department of Human Anatomy and Embryology Faculty of Medicine University of Granada Granada 18016 Spain; ^4^ BioFab i3D Lab – Biofabrication and 3D (bio)printing Laboratory Granada 18016 Spain; ^5^ Excellence Research Unit “Modeling Nature” (MNat) University of Granada Granada Spain; ^6^ Center of Advanced Scientific Research (CICA) University of La Coruña A Coruña 15001 Spain; ^7^ Complex Tissue Regeneration Department MERLN Institute for Technology‐Inspired Regenerative Medicine Maastricht University Universiteitssingel 40, ER Maastricht 6229 The Netherlands; ^8^ F2N2Lab Magnetic Soft Matter Group, Department of Applied Physics Faculty of Sciences University of Granada Granada 18003 Spain; ^9^ R&D Human Health, Bioibérica S.A.U. Barcelona 08950 Spain; ^10^ Institute of Neuroscience Center for Biomedical Research (CIBM) University of Granada Granada 18016 Spain; ^11^ Animal Behavior Research Unit Scientific Instrumentation Center University of Granada Granada 18003 Spain; ^12^ Department of Health Sciences University of Jaén Jaén 23071 Spain

**Keywords:** biofabrication, bioinks, fibrin, skin, sprays

## Abstract

In the last years, different biofabrication methods have gained special attention for the production of skin substitutes that overcome the limitations of conventional skin grafting. Skin sprays represent a promising technology for treating cutaneous wounds as they can deliver both cells and biomaterials to the wound bed in a fast and easy approach, covering extensive wound surfaces. The aim of this study is to develop a novel bioink based on fibrinogen supplemented with a glycosaminoglycans (GAGs)/collagen (Col)‐based matrix, containing hyaluronic acid, dermatan sulfate, chondroitin sulfate, and Col, in combination with an innovative dual‐head airbrush‐based spraying device. The fibrinogen/GAGs/Col‐based bioink is loaded with human mesenchymal stromal cells or human dermal fibroblasts, and its physicochemical and mechanical properties are analyzed, as well as cell viability, metabolic activity, and in vitro wound healing. Finally, its skin wound healing properties are studied in an in vivo excisional wound healing murine model. The bioink forms hydrogels with satisfactory physicochemical, mechanical, and biological properties, capable of promoting wound healing and tissue regeneration in vivo with outcomes comparable to those of autografts. The novel spray system and bioink show the potential to serve as a therapeutic tool for the clinical treatment of cutaneous wounds.

## Graphical abstract

A dual‐head airbrush‐based spraying device, combined with a fibrinogen bioink enriched with glycosaminoglycans and collagen, enables the delivery of biomaterials and cells to promote wound healing. This system demonstrates effective skin regeneration in vitro and in vivo, with comparable results to autografts. The innovative spray approach offers a minimally invasive, scalable solution for the clinical treatment of cutaneous wounds.

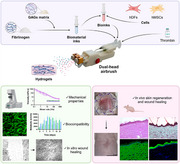

## 1 Introduction

Skin is one of the most important defense mechanisms of the body, protecting it against pathogens, chemicals, and physical hazards. It plays other essential roles, such as controlling the body's temperature and moisture, preventing transepidermal water loss (TEWL), or perceiving pain, touch, and temperature, among other functions.^[^ ^1^, ^2^, ^3^ ^]^ However, its direct contact with the external environment makes it highly vulnerable to being injured. Shallow injuries can usually be naturally regenerated by the skin's self‐healing functions, but in deep wounds, the skin's regenerative elements are destroyed, requiring therapeutic interventions to facilitate wound healing.^[^ ^2^, ^4^ ^]^

Traditionally, the gold standard in clinical practice for treating deep wounds has been the use of autologous skin grafts (autografts). Despite their effectiveness, autografts present important limitations, such as the unavailability of enough donor skin in cases like major burn patients and the creation of a secondary wound for the patient. Other grafting options include the culture of cells harvested from a small donor site, which requires weeks to amplify their number, or the temporary use of allografts or xenografts, which have the risk of disease transmission and/or immune rejection.^[^ ^5^, ^6^, ^7^ ^]^

However, thanks to the advances in regenerative medicine and tissue engineering, different biofabrication methods like 3D bioprinting or electrospinning have been developed for the production of skin substitutes.^[^ ^8^, ^9^, ^10^, ^11^ ^]^ Those techniques generally use biomaterial inks, which are acellular formulations containing biologically active components or molecules (but that can be subsequently seeded with cells afterward), or bioinks, cell‐based formulations that may also contain biomaterials and other biologically active components.^[^ ^12^ ^]^ A promising biofabrication method with application in skin regeneration is the use of skin sprays. Although this technology has not been extensively exploited in the published literature yet, it presents several advantages for the delivery of bioinks or biomaterial inks, which include the possibility of rapidly treating large areas with unfavorable topography, generating a homogeneous distribution of the sprayed material.^[^ ^13^ ^]^

Several acellular spray products have been commercialized for decades now, the majority of them based on fibrin due to its hemostatic properties.^[^ ^14^ ^]^ For example, TISSEEL and ARTISS by Baxter,^[^ ^15^, ^16^ ^]^ VISTASEAL by Johnson & Johnson,^[^ ^17^ ^]^ or Vivostat's Fibrin Sealant.^[^ ^18^ ^]^ The spraying devices of these fibrin sealant products are generally double syringes (one syringe for fibrinogen and another for thrombin, its crosslinker) with a spray applicator tip; except for Vivostat, which offers the Spraypen, a hand‐held disposable device for the delivery of their Fibrin Sealant.^[^ ^19^ ^]^ Although these devices are intended for the use of their fibrin sealants, it would be of interest to have the possibility to deliver different biomaterials. Acellular spray products based on fibrin may simplify clinical translation by avoiding regulatory complexities associated with cell‐based products.^[^ ^13^ ^]^ However, incorporating cells into the ink product enhances its therapeutic potential by providing both structural support and biological activity via the secretion of cytokines and growth factors that promote tissue regeneration.^[^ ^11^, ^20^ ^]^ Various cell types have been employed in cell spray techniques for in vitro wound healing studies. Among them, autologous epidermal cells remain the primary choice for in vivo studies and clinical trials. However, in extensive wounds, donor sites may be limited; thus, allogeneic cells may be used, provided they do not trigger an immune response or risk of rejection. Cell spray technology represents a novel therapeutic approach currently under clinical investigation, with only a limited number of products having obtained regulatory approval for commercialization and clinical application.^[^ ^13^ ^]^ The ReCell kit by Avita Medical was the first “*spray‐on‐skin*” treatment approved by the U.S. Food and Drug Administration (FDA) in 2018, which employs an enzyme solution to isolate autologous epidermal cells from a small biopsy of the patient's healthy skin. Then, they are suspended in a lactate solution and sprayed over the wound using a syringe with a spray nozzle.^[^ ^21^, ^22^, ^23^, ^24^, ^25^ ^]^ However, the use of syringes does not allow the control of essential spraying parameters such as the spraying pressure, which is crucial for obtaining optimal cell viability.^[^ ^3^ ^]^ Moreover, cell suspensions can usually run off when sprayed over the body's convex structures, reducing the number of cells that stay in contact with the wound bed. To avoid this problem and ensure that cells adhere to the wound, cell suspensions are usually sprayed in combination with biomaterials, such as fibrinogen, which increases the viscosity of the solution, and once crosslinked, forms a solidified fibrin matrix that remains attached to the wound surface.^[^ ^26^, ^27^, ^28^ ^]^

Fibrin hydrogels are highly biocompatible and play a vital role in wound healing and hemostasis. Fibrin is obtained during the coagulation cascade when fibrinogen is converted into a fibrin clot by the action of thrombin. It promotes cell migration toward the wound site and forms a matrix where cells can proliferate and promote tissue regeneration.^[^ ^29^, ^30^, ^31^, ^32^ ^]^ However, the performance of fibrin hydrogels can be further enhanced by incorporating extracellular matrix (ECM) constituents. ECM molecules, such as glycosaminoglycans (GAGs), which include hyaluronic acid (HA), heparan sulfate, dermatan sulfate, chondroitin sulfate, and keratan sulfate, play crucial roles in all stages of wound healing. HA, the skin's predominant GAG, has anti‐inflammatory and mucoadhesive properties. Furthermore, it has a high water retention capability, which defines skin elasticity and helps to inhibit scar tissue formation.^[^ ^33^, ^34^, ^35^, ^36^, ^37^, ^38^, ^39^ ^]^ In addition, both GAGs and collagen (Col) have also been shown to promote cell proliferation and migration and the synthesis of matrix components such as Col or elastin.^[^ ^40^, ^41^, ^42^, ^43^, ^44^, ^45^, ^46^ ^]^

In the present study, we have developed a novel dual‐head spray device together with a fibrinogen/GAGs/Col‐based bioink for application in the treatment of skin lesions. The spraying device was based on an airbrush with a modified design to spray two different solutions at the same time. The bioink was based on a blend of fibrinogen and a GAGs/Col‐based matrix with a high concentration of HA, and was compared to a fibrinogen‐only bioink. Both bioinks were loaded with human mesenchymal stromal cells (hMSCs) and human dermal fibroblasts (hDFs), and their physicochemical and mechanical properties, as well as their in vitro biocompatibility, were analyzed. Finally, their skin wound healing properties were studied in an in vivo excisional wound healing murine model.

## 2 Experimental Section

### 2.1 Cell Culture

hMSCs and hDFs were isolated from human adipose tissue and skin samples, respectively, obtained from abdominoplasty procedures. The samples were collected at the *Vithas Hospital Granada (Plastic Surgery Service)* with informed consent and Institutional Review Board approval (ethics committee number: 0467‐N‐20) covering both tissue types, and characterized as previously reported.^[^ ^47^, ^48^, ^49^ ^]^ hMSCs and hDFs were cultured in high‐glucose Dulbecco's Modified Eagle's Medium (DMEM; Sigma‐Aldrich) supplemented with 10% fetal bovine serum (FBS) and 1% penicillin and streptomycin (P/S; Invitrogen). Cultures were maintained at 37 °C in a humidified atmosphere containing 5% CO_2_. The medium was changed every 2—3 days, and cells were sub‐cultured at ≈80% confluence. For all the experiments, cells were used between passages 6 and 8.

### 2.2 Formulation of Fibrinogen‐Based Bioinks

The novel bioink consisted of fibrinogen supplemented with a rooster comb‐derived GAGs/Col‐based matrix composed of a high concentration of HA (67%), sulfated GAGs (12%, including dermatan sulfate and chondroitin sulfate), and Col (10.4%) (Dermial, Bioibérica S.A.U., Barcelona, Spain),^[^ ^46^, ^50^ ^]^ and it was compared to a fibrinogen‐only bioink. To prepare the (acellular) fibrinogen (Fib) biomaterial ink (from now on referred to as “ink”), first bovine fibrinogen (Sigma‐Aldrich) was dissolved in a 0.9% NaCl solution at 37 °C to obtain a fibrinogen concentration of 10 mg mL^−1^. Once dissolved, the solution was filter‐sterilized with 0.22 µm membrane filters (Merck Millipore). Then, for the fibrinogen‐Dermial (FibD) ink, previously UV‐sterilized Dermial powder was added to the Fib ink at a concentration of 2.5 mg mL^−1^. The inks were used immediately after preparation or stored at 4 °C for no longer than 24 h before use. To prepare the cell‐loaded bioinks, hMSCs or hDFs were then gently mixed with the Fib or FibD inks at a cell density of 1 × 10^6^ cells mL^−1^. Once resuspended, the cell‐loaded bioinks were typically used immediately or within ≈2 h, at room temperature and under sterile conditions.

Additionally, bovine thrombin (Sigma‐Aldrich) was diluted in 40 mm CaCl_2_ at a final concentration of 50 U mL^−1^, and filter‐sterilized. Finally, the fibrin‐based hydrogels were obtained by mixing the Fib or FibD inks or bioinks with the thrombin solution in a 10:1 (v/v) ratio, and letting them gel for at least 5 min at 37 °C.

All components (fibrinogen, GAGs/Col matrix, thrombin, and cells) were used at the previously indicated concentrations and ratios in all experiments to ensure reproducibility, with volumes adjusted as needed depending on the specific requirements of each assay.

### 2.3 Spraying Device Design and Performance

The hand‐held spraying device was based on an airbrush with a modified design to support dual dispensing. The device comprised a 3D‐printed chassis encasing a double spray system and electronic connections. The chassis was designed using Inventor 3D CAD software (Autodesk Inc.), and 3D printed with a Raise3D Pro Plus (Raise3D, USA) printer, using acrylonitrile butadiene styrene (ABS, Raise3D), due to its good mechanical properties, durability, and toughness. The double spray system consisted of two external disposable syringes: the Fib or FibD inks or bioinks were loaded in one syringe, while the thrombin solution was loaded in the other. Both syringes were connected to their respective spray nozzles. The pressurized air inlet came from an air pump (HABA Trading B.V., The Netherlands) and passed through a 0.22 µm membrane filtered to ensure air sterility. Before use and between different experiments, the syringes, internal conduits, and nozzles were carefully cleaned by rinsing with ethanol, followed by a phosphate‐buffered saline (PBS) wash. All experiments were conducted in a laminar flow hood to guarantee sterility.

In all assays, the angle between the spraying device and the target surface was kept at 45°, with a separating distance of ≈10 cm. For the in vitro and in vivo assays, an acetate‐derived plastic cone was used as a confining funnel to focalize all the sprayed material into the wells of the culture plates or the wounds on mice, and therefore, avoid sprayed volume loss.

#### 2.3.1 Optimization of the Spraying Pressure

To optimize the best spraying pressure, the metabolic activity (an indicator that could be relatively related to cell proliferation) of hDFs in the FibD bioink after spraying at different pressures (10, 15, 20, and 30 psi) was analyzed with an AlamarBlue HS assay (Invitrogen, CA, USA). For each sample, 500 µL of bioink and 50 µL of thrombin solution were sprayed in 24‐well plates and left to gel for 5 min. Briefly, after 1, 2, 3, 5, and 7 days of culture, hydrogel samples were incubated with 500 µL of a 10% AlamarBlue HS solution at 37 °C for 1 h. Then, fluorescence intensity was measured at 530/590 nm excitation/emission wavelengths in a Synergy HT multidetection microplate reader (BioTek Instruments, Inc., Winooski, VT, USA).

#### 2.3.2 Spray Characterization: Cone Angle and Droplet Size

To characterize the performance of the spraying device, the spray cone angle and droplet size distribution were analyzed using visual and image‐based approaches.

The spray cone angle was assessed from both top and lateral views by spraying with the device, at 15 psi, against a black background under ambient lighting. Photographs were taken using a smartphone camera after activating each spray mode (left nozzle, right nozzle, and dual spray), and the angle was calculated by overlaying dashed lines over the visible spray edges and measuring the resulting angle using a protractor tool.

To evaluate the droplet size distribution, a single short burst (≈0.5 s) of bioink was sprayed at 15 psi, at a 10 cm distance, onto a clean parafilm sheet to minimize droplet overlap, in triplicate. A Leica S9i digital stereo microscope (Leica Microsystems GmbH, Germany) at 5× magnification was used to capture images of the deposited droplets, with the Leica Application Suite (LAS) V4.12 software. Images were then analyzed with ImageJ (Fiji) software to obtain droplet size.

### 2.4 Physicochemical Properties

#### 2.4.1 pH Determination

The pH of the Fib and FibD inks was measured (*n* = 6) with a calibrated digital pH‐meter Hach Sension+ (Hach Lange S.L., Spain) at room temperature.

#### 2.4.2 Swelling Test of Fib and FibD Hydrogels

Previously freeze‐dried hydrogels were weighed, submerged in PBS, and kept at 37 °C without agitation. At determined time points (0, 6, 24, 48, and 72 h), samples (*n* = 6) were taken out, absorbing excess of PBS with filter paper, and weighed. The swelling rates (%) were calculated using the following equation:

(1) $$Swelling\;ratio\left(\%\right)=\left(\frac{W_{t}-W_{0}}{W_{0}}\right)\times 100$$

where *W*_0_ is the initial wet weight of the samples at the initial time (*t* = 0 *h*), and *W_t_* is the wet weight of the samples at a later point (*t*).

#### 2.4.3 Degradation Test of Fib and FibD Hydrogels

Pre‐weighed hydrogels were immersed in PBS and maintained at 37 °C under gentle agitation. At determined time intervals (0 h, 6 h, days 1, 2, 3, 5, and 7, and then weekly for 20 weeks), samples (*n* = 6) were centrifuged at 3000 g for 10 min, the supernatant was removed, and hydrogels were weighed. The degradation rates (%) were calculated with the following equation:

(2) $$Degradation\;ratio\;\left(\%\right)=\left(\frac{W_{0}-W_{t}}{W_{0}}\right)\times 100$$

where *W*_0_ is the initial wet weight of the samples at the initial time (*t* = 0 *h*), and *W_t_* is the wet weight of the samples at a later time (*t*).

#### 2.4.4 Mechanical and Rheological Characterization

Rheological assays were carried out to measure the viscosity curves of the fibrinogen‐based inks, as well as the compression (Young's modulus) and shearing (viscoelastic moduli) characteristics of the hydrogels, using a torsional rheometer MCR302 (Anton Paar, Austria). All the rheological assays were performed in triplicate in isothermal conditions at 25 °C.

The shear viscosity of the Fib and FibD inks was obtained using a cone‐plate configuration (50 mm diameter and 1° angle). As a reference, distilled water and NaCl 0.9% solutions were also measured. First, they were pre‐sheared at a constant shear rate of 800 s^−1^ for 1 min to remove the mechanical history of the sample. Then, they were allowed to rest for 1 min, with no shear rate applied. Finally, they were continuously sheared following a logarithmic shear rate ramp from 0.01 to 800 s^−1^ for 5 min, with an acquisition time of 5 s.

The compression and shearing characteristics of the hydrogels were measured using a plate–plate configuration (20 mm diameter). For this aim, the hydrogels were fabricated in cylindrical molds of 20 mm diameter and 5 mm height, and the mice skin samples were cut into circles with 20 mm diameter. A typical experiment comprised three intervals. In the first interval, samples were gently compressed by the upper tool of the rheometer at a constant approaching speed of 10 µm^−1 ^s, up to a normal force of 0.1 N (in the case of the hydrogels) or 0.5 N (in the case of the mice skin), to determine the Young's modulus. In the second interval, the normal force was kept constant for 30 s (either at 0.1 N or 0.5 N, depending on the sample), for stabilization. Finally, in the third interval, the samples were oscillatory sheared with a logarithmically increasing strain amplitude from 0.001% to 1000%, at a strain frequency of 1 Hz and a constant normal force (0.1 N for hydrogels or 0.5 N for mice skin) to determine the shear viscoelastic moduli (i.e., storage and loss moduli).

### 2.5 Biological Characterization

#### 2.5.1 Cell Viability and Metabolic Activity

The metabolic activity of hDFs and hMSCs, as a relative indicator of cell proliferation, was analyzed with the AlamarBlue HS assay after 0, 1, 3, 5, 7, 14, and 21 days of culture, as described previously.

The LIVE/DEAD Viability/Cytotoxicity Kit (Invitrogen Inc., Grand Island, NY, USA) was used to assess the cell viability of hDFs and hMSCs after 1, 7, 14, and 21 days of culture. For each sample, 500 µL of bioink and 50 µL of thrombin solution were sprayed in 24‐well plates. Samples were stained with 500 µL of calcein AM (2 µm) and ethidium homodimer I (EthD‐I; 4 µM) solutions in PBS at 37 °C for 30 min. Then, they were observed using a confocal microscope (Leica TCS‐SP5 II DMI6000B, Leica Microsystems), showing viable cells green (calcein AM) and non‐viable cells red (EthD‐I), with laser settings at 494/517 nm for calcein AM and 528/617 nm for EthD‐I. Images were analyzed with the ImageJ (Fiji) software,^[^ ^51^ ^]^ quantifying live and dead cells, and determining the percentage of cell viability with the following equation:

(3) $$Cell\;viability\left(\%\right)=\left(\frac{Live\;cells}{Live+dead\;cells}\right)\times 100$$

#### 2.5.2 Scratch Wound Healing Assay

hDFs and hMSCs were seeded in 6‐well culture plates at a density of 1.5 × 10^5^ and 2 × 10^5^ cells well^−1^, respectively. Once they reached 80% confluency, the culture medium was removed, and monolayers were “wounded” by manual scratching with a 200 µL pipette tip, making two parallel scratches per well, in a standardized and consistent manner by the same operator, following a uniform direction and angle, to minimize variability between samples. Then, monolayers were gently washed with PBS to remove cell debris, and the corresponding media for each condition were added to each well (*n* = 6 per condition): 1) Control (culture medium, that is, DMEM supplemented with 10% FBS and 1% P/S); 2) Fib (culture medium supplemented with fibrinogen at 10 mg mL^−1^); 3) Dermial (culture medium supplemented with Dermial at 2.5 mg mL^−1^); and 4) FibD (culture medium supplemented with fibrinogen at 10 mg mL^−1^ + Dermial at 2.5 mg mL^−1^).

Images of the wounds were taken at 0, 12, 24, and 48 h after scratching, using a Leica DMi8 microscope (Leica Microsystems) with the Leica Application Suite (LAS) X software. Wound areas at each time point were measured with the ImageJ software, and the migration rate (expressed as the percentage of area reduction or wound closure) was calculated with the following equation:^[^ ^52^ ^]^

(4) $$Wound\;closure\left(\%\right)=\left(\frac{A_{t_{0}}-A_{t_{h}}}{A_{t_{0}}}\right)\times 100$$

where $A_{t_{0}}$ is the area of the wound measured immediately after scratching (*t* = 0 *h*) and $A_{t_{h}}$ is the area of the wound measured *h* hours after the scratch is performed.

### 2.6 In Vivo Wound Healing Assay

#### 2.6.1 Wound Healing Murine Model, Surgical Procedures, and Experimental Groups

**Figure 1:**
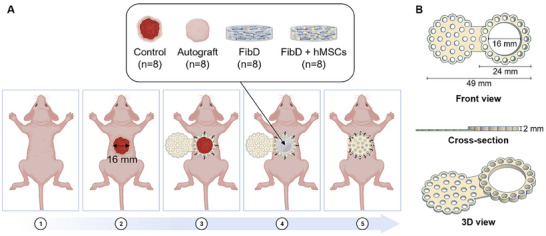
A) In vivo excisional wound healing assay surgical protocol. B) b‐TPUe splint design. Figure created with BioRender.com.

A total of 32 ATHYM‐Foxn1^nu/nu^ male and female, immunodeficient, athymic, nude, and albino mice (Janvier Labs, Le Genest‐Saint‐Isle, France) of 7 weeks of life, were employed for the in vivo assay. Animals were randomly assigned to 4 groups (*n* = 8 per group): 1) Control; 2) Autograft; 3) FibD; and 4) FibD + hMSCs (**Figure** **1**A). All animal handling procedures complied with the national and European Union legislation (Spanish RD 53/2013 and EU Directive 2010/63) for the protection of animals used for scientific purposes and following the Ethical Principles and Guidelines for the Use of Animals approved by Provincial Ethics Committees of Granada (reference number: 1/06/2022/081).

Mice were anesthetized using isoflurane inhalation (Isoflurane 1000 mg mL^−1^; Fatro Ibérica, Desvern, Spain), and a round full‐thickness skin area of 2 cm^2^ was removed from the upper dorsal, in a longitudinal position to the mouse spine, using surgical scissors. To prevent mice from scratching or biting the wounds/hydrogels and avoid wound contraction, sterile 3D printed 1,4‐butanediol thermoplastic polyurethane elastomer (b‐TPUe; Filaflex 80A, Recreus Industries S.L., Spain)^[^ ^53^ ^]^ splints were anchored to the wound edges with seven interrupted nylon sutures. The b‐TPUe splint was designed porous and toroidal, with a hinged lid, and 3D printed with a Regemat 3D Bio V1 bioprinter (REGEMAT 3D S.L., Spain) (Figure 1B).

The Control group was left untreated, with only the b‐TPUe splint; while in the case of the Autograft group, another round full‐thickness skin area of 2 cm^2^ was removed from the lower back of the mice and grafted over the first wound. Then, this second wound was closed by suturing the wound's edges together. For the sprayed hydrogels, the FibD ink or FibD + hMSCs (1 × 10^6^ hMSCs mL^−1^) bioink, and thrombin solutions were sprayed simultaneously over the wound bed and were left to crosslink for ≈5 min.

For all groups, the b‐TPUe splint lid was closed with eight interrupted sutures, and an antibiotic ointment (Mupirocina 20 mg g^−1^; ISDIN, Spain) was applied. Then, analgesic (Bupredine 0.3 mg mL^−1^, 10 mL; Fatro Ibérica, Desvern, Spain) and antibiotic (Ganadexil Enrofloxacin 5%, 100 mL; Industrial Veterinaria, S.A. Invesa, Spain) solutions were subcutaneously injected for the next 3 days as postoperative treatment. The splints were maintained in place for the first 14 days to prevent wound contraction during the early healing phase. On day 14, they were carefully removed under brief isoflurane anesthesia prior to performing wound area measurements and assessments of wound homeostasis.

#### 2.6.2 Skin Repair Monitoring

An exhaustive clinical follow‐up of skin repair was carried out for 8 weeks, assessing macroscopic wound healing and measuring the scar/wound area with the ImageJ software. As well, an adaptation of the patient and observer scar assessment scale (POSAS)^[^ ^54^, ^55^ ^]^ was used to evaluate scars after 8 weeks.

Moreover, every 2 weeks, mice were anesthetized by isoflurane inhalation, and several skin homeostasis parameters were measured using the Microcaya probe system (Microcaya S.L., Spain): the Thermometer probe measured skin temperature (°C); the Skin pH‐meter probe determined skin pH; the Tewameter probe measured TEWL as the evaporation of water (g h^−1^ m^−2^); the Corneometer probe determined moisturization through the capacitance of a dielectric medium (in arbitrary units, AU); the Cutometer probe evaluated elasticity (µm) with suction (450 mbar of negative pressure −2 s); and the Mexameter probe obtained information about skin pigmentation (melanin) and vascularization (hemoglobin levels) based on light absorption/reflection of three wavelengths, and thereby measuring pigmentation and erythema (in AU). All of these parameters were measured on both scars/wounds and areas of healthy skin (from the lower back region, as healthy skin control measurements) from each mouse in the study.

#### 2.6.3 Histology and Immunofluorescence

After 4 and 8 weeks, respectively, half of the mice of each group were anesthetized with isoflurane inhalation to harvest the regenerated tissue samples and the surrounding skin, and subsequently euthanized by cervical dislocation. Healthy skin samples were also taken from different mice at 4 and 8 weeks. The obtained samples were fixed in 4% paraformaldehyde and dehydrated for histology and immunofluorescence.

For the histological analysis of the regenerated skin, fixed samples were embedded in paraffin and cut into 8 µm sections using a microtome. The sections were dewaxed, rehydrated, and stained with hematoxylin and eosin (H&E) and with Masson's Trichrome (MT), following the manufacturer's instructions.

For the immunofluorescence analysis, fixed samples were embedded in optimal cutting temperature compound (Tissue‐Tek, Sakura Finetek) before frozen sectioning on a cryostat. The 8 µm sections were blocked in 2% Blocking Solution (Blocking Reagent, cat. 11 096 176 001, Roche; prepared in maleic acid buffer, then diluted in PBS) and subsequently incubated with the following primary antibodies: pan Cytokeratin (CK, Invitrogen, dilution 1:100), Col I (Abcam, dilution 1:200), and Fibronectin (FN, Santa Cruz Biotechnology, dilution 1:200). Then, the sections were washed and incubated with secondary Alexa‐488‐conjugated antibody (ThermoFisher, dilution 1:500). Finally, nuclei were counterstained with DAPI (dilution 1:1000). Sections were washed and covered with glass coverslips fixed with Fluoroshield mounting medium (Sigma‐Aldrich).

Standard light and fluorescence images for histology and immunofluorescence, respectively, were obtained with a Leica DMi8 microscope.

### 2.7 Statistical Analysis

All graphed data were represented as mean ± standard deviation (SD). Statistical calculations were performed using GraphPad Prism 8.0.1 software. Data were analyzed using parametric or non‐parametric tests depending on the distribution and homogeneity of variances, as assessed by the Shapiro–Wilk and Brown–Forsythe tests, respectively. For comparisons between groups, one‐way or two‐way ANOVA tests, or mixed‐effects model with the Geisser–Greenhouse correction, were performed with appropriate post‐hoc analyses (e.g., Tukey, Dunnett, Tamhane T2, or Sidak,) for multiple comparisons. Non‐parametric tests, such as Kruskal–Wallis with Dunn's post‐hoc test, were applied where data did not meet normality or homoscedasticity assumptions. Specific tests for each experiment are detailed in the figure legends. Differences were considered statistically significant at *p* < 0.05 (*/#), *p* < 0.01 (**/##) and *p* < 0.005 (***/###).

## 3 Results

### 3.1 Spraying Device

**Figure 2:**
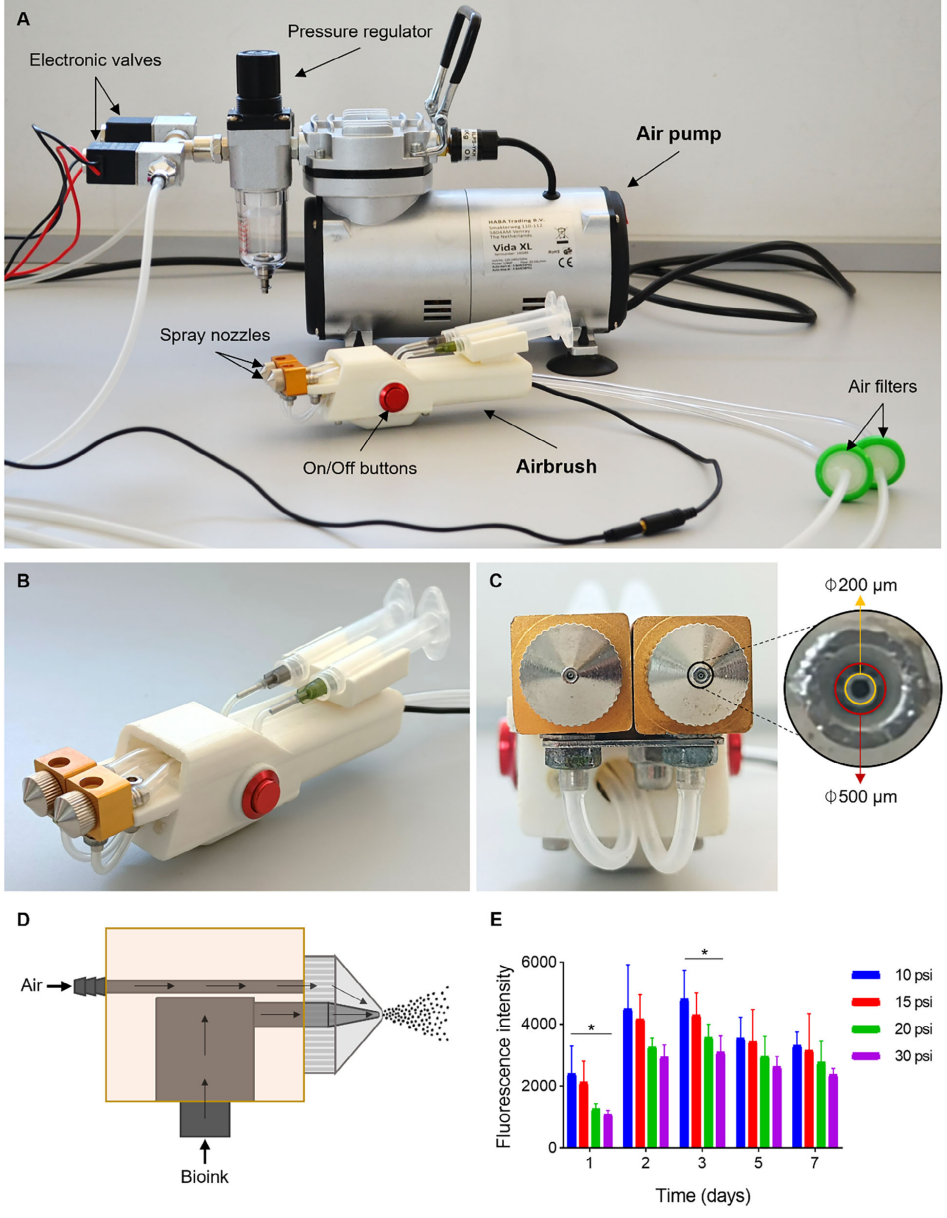
Spraying system. A) Overview. B) Airbrush. C) Airbrush front view and nozzle diameter. D) Schematic representation of the spray nozzles. E) Metabolic activity, measured as fluorescence intensity (AU), of hDFs sprayed at different pressures (*n* = 4 per condition) in FibD hydrogels at days 1, 2, 3, 5, and 7. Statistical analysis was performed using an ANOVA test, followed by Tukey's post‐hoc test. Statistical significance: *p < 0.05.

The airbrush‐based spraying device was designed to support dual dispensing, controlled by a pneumatic system (**Figure** **2**; Figure S1, Supporting Information). Placed on each side of the device, two On/Off buttons (one for each material) connected to electronic valves open/close the airflow inlet from the air pump (Figure 2A). By pressing one of the On/Off buttons or both at the same time, both solutions can be sprayed either subsequently or simultaneously, respectively (Figure 2B). A pressure regulator placed at the air pump controls air pressure, and consequently, the material deposition rate. By increasing the air pressure, more shear is exerted, and air more strongly pushes the material out through the nozzle. The material is extruded through an inner 200 µm‐diameter nozzle, while air is pushed through an outer 500 µm‐diameter nozzle (Figure 2C). When the material meets the air stream near the outlet orifice of the nozzles, the material is pulverized generating bioink droplets (Figure 2D). The concentration of the sprayed material is presumed to remain consistent, as the entire volume was dispensed during each spraying event without dilution. The dimensions of the device, as well as the internal design, are depicted in Figure S1 (Supporting Information).

An AlamarBlue HS assay was used to determine the best spraying pressure for our bioink. The metabolic activity of hDFs decreased with increasing spraying pressure, showing evidence of how cells are damaged with increasing pressures (Figure 2E). Although the lowest pressure (10 psi) showed the highest metabolic levels, this pressure was not strong enough to maintain a constant and steady flow of the sprayed material. Therefore, a spraying pressure of 15 psi was chosen for the following experiments as it still showed high metabolic activity levels, close to those of 10 psi.

To further characterize the spray behavior, the cone angle and droplet size were analyzed at the selected working pressure of 15 psi. From top and side views, the spray cone angle for each nozzle was ≈16° and 12°, respectively (Figure S2A,B, Supporting Information). Droplet deposition was evaluated by briefly spraying onto a parafilm sheet in 3 independent trials. The deposition pattern (Figure S2C, Supporting Information) showed well‐separated droplets, suggesting adequate pulverization of the bioink at this pressure. Image analysis of the 3 replicates (*n* = 291, 224, and 253 droplets, respectively; 768 droplets in total) revealed a broad size distribution, with most droplets (75.91%) being below 100 µm in diameter. Mean droplet sizes were similar across replicates (67.5 ± 62 µm, 65.7 ± 47.7 µm, and 61.4 ± 38.7 µm for n1, n2, and n3, respectively), with an overall mean diameter of 64.98 ± 51.13 µm (*n* = 768 droplets). Droplet sizes ranged from 6.3 to 269.4 µm, 4.4 to 242 µm, and 7 to 188.8 µm for each replicate, respectively (Figure S2D, Supporting Information).

### 3.2 Physicochemical Properties

#### 3.2.1 pH, Swelling, and Degradation Performance

**Figure 3:**
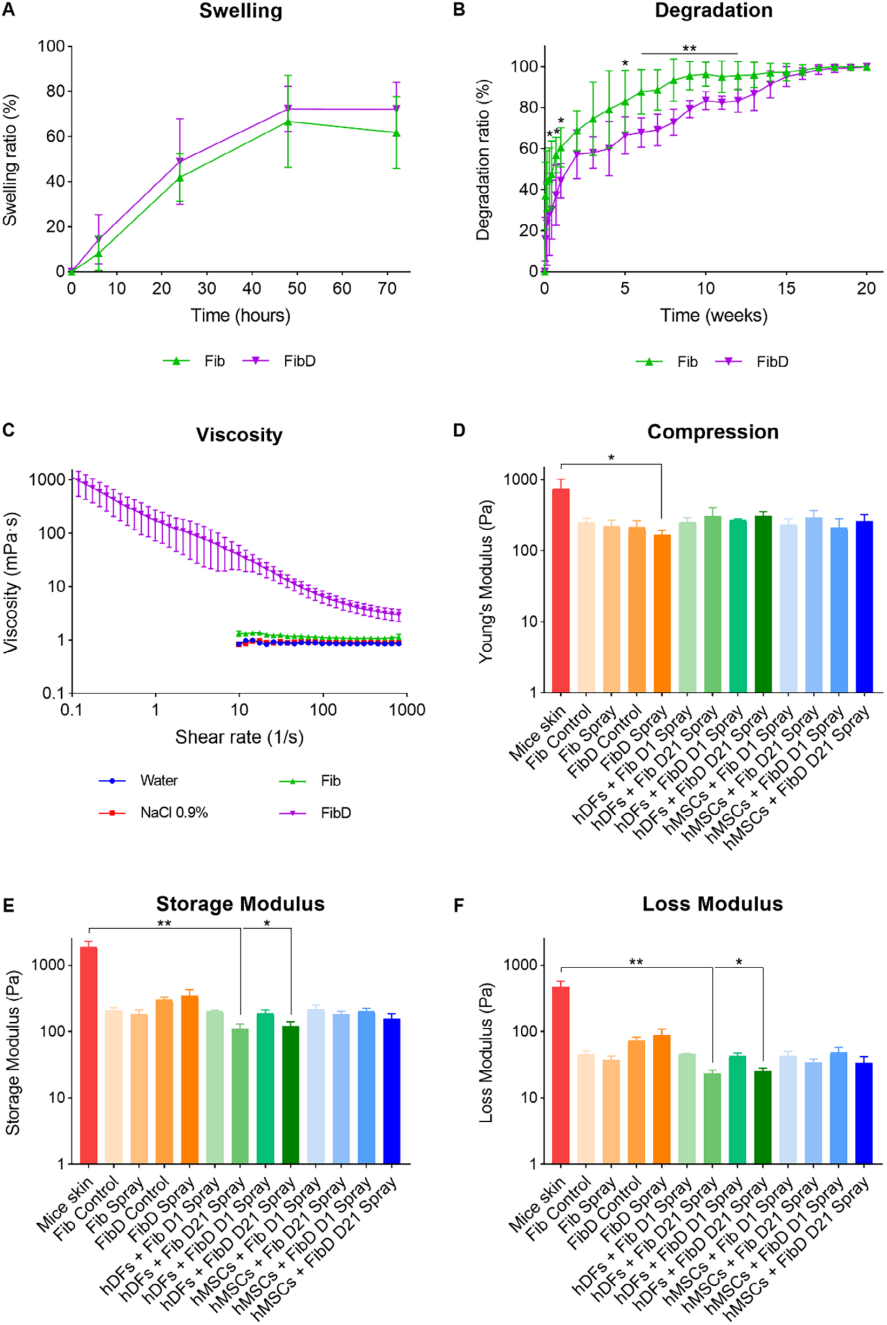
Physicochemical, rheological, and mechanical characterization. A) Swelling (*n* = 6 per condition) and B) Degradation ratio (*n* = 6 per condition) of Fib and FibD hydrogels. C) Viscosity of distilled water, 0.9% NaCl, and Fib and FibD inks (*n* = 3 per condition). D) Young's Modulus; and viscoelastic moduli [E) Storage Modulus and F) Loss Modulus], of mice skin and hydrogels (*n* = 3 per condition). Statistical analysis for Swelling and Degradation was conducted using a mixed‐effects model with the Geisser–Greenhouse correction, followed by Sidak's post‐hoc test. For Young's Modulus and Viscoelastic moduli, the Kruskal–Wallis test was applied, followed by Dunn's post‐hoc test. Statistical significance: *p < 0.05; **p < 0.01; ***p < 0.005.

The pH values of the Fib and FibD inks were 6.12 ± 0.19 and 6.1 ± 0.17, respectively. The swelling behavior of previously freeze‐dried Fib and FibD crosslinked hydrogels was observed by immersing the samples in PBS and measuring their weight increase (**Figure** **3**A). For both Fib and FibD hydrogels, the swelling ratio increased during the first 48 h, reaching an average swelling of 66.7 ± 10.2% and 72.2 ± 5% for Fib and FibD, respectively. Afterward, the hydrogels’ weight started to decrease slightly due to degradation. The swelling ratio of the FibD hydrogels was slightly higher than Fib at all time points, but no significant differences were observed. On the other hand, the degradation rate of hydrogels was analyzed by measuring weight loss over time (Figure 3B). The degradation of Fib and FibD hydrogels increased rapidly during the first week and then kept increasing moderately every week. The Fib hydrogels degraded slightly faster than the FibD hydrogels, presenting a higher degradation ratio during the experiment, with significant differences at 6 h, days 5 and 7, and weeks 5 to 12. All Fib hydrogels had degraded completely after 17 weeks, whereas FibD hydrogels degraded after 20 weeks.

#### 3.2.2 Mechanical and Rheological Characterization

To assess the capability of the bioinks to be sprayed through the airbrush's nozzle, the shear viscosity of the Fib and FibD inks was determined under a steady shearing flow. As a reference, the viscosity of distilled water and 0.9% NaCl (the basis of the fibrinogen‐based inks formulation) was also measured. The viscosity curves of all materials tested are shown in Figure 3C at 25 °C. The viscosity of water and NaCl solutions was 0.871 ± 0.039 and 0.896 ± 0.272 mPa s, respectively. As expected, they demonstrated a Newtonian flow behavior, with the viscosity being independent of the shear rate. Interestingly, the Fib ink also showed a Newtonian behavior, with a constant viscosity of 1.163 ± 0.095 mPa s, while the addition of the GAGs/Col matrix to the FibD ink produced a strongly shear‐thinning behavior, with a decreasing viscosity with increasing shear rates (ranging from 1114.75 mPa s at $\dot{\gamma }$ = 0.1 s^−1^, to 2.97 mPa s at $\dot{\gamma }$ = 800 s^−1^).

Moreover, the compression and viscoelastic characteristics of the hydrogels formed from the bioinks were also analyzed (Figure 3D–F). Manually created (pipetted) Fib and FibD hydrogels (Control) were compared with sprayed Fib and FibD hydrogels, respectively, with no significant differences. Differences between Fib and FibD hydrogels were not statistically significant, but higher storage (Figure 3E) and loss (Figure 3F) moduli were observed for the FibD hydrogels than the Fib ones. This difference was not found in cell‐loaded hydrogels. For hDFs and hMSCs‐loaded samples, both with Fib and FibD, hydrogels cultured for 21 days showed higher Young's moduli (Figure 3D) and lower viscoelastic moduli (Figure 3E,F), than those cultured for 1 day, although differences were not statistically significant. Finally, samples of healthy skin of mice revealed higher Young's and viscoelastic moduli than all hydrogels, although differences were only statistically significant with the sprayed FibD hydrogels for the Young's Modulus (Figure 3D), and with the sprayed hDFs and hMSCs‐loaded FibD hydrogels after 21 days for the viscoelastic moduli (Figure 3E,F).

### 3.3 Biological Characterization

#### 3.3.1 Cell Viability and Metabolic Activity

The biocompatibility of the Fib and FibD bioinks was analyzed by cell metabolic activity (used as a relative indicator of cell proliferation) and viability assays. Hydrogels were created by simultaneously spraying the thrombin solution and the Fib and FibD bioinks loaded with hDFs or hMSCs. On the other hand, hydrogels created manually (pipetted) were used as controls.

**Figure 4:**
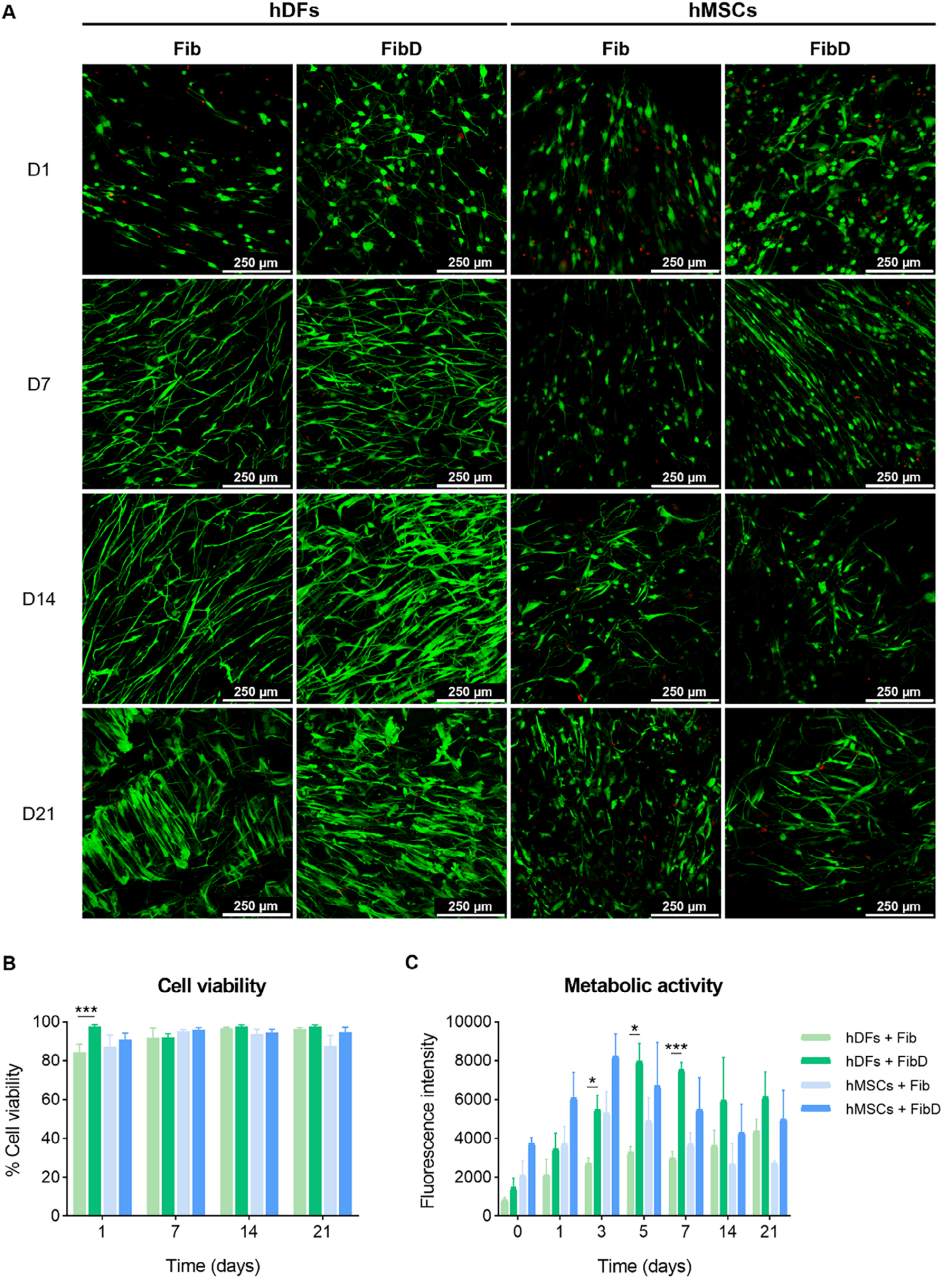
Cell viability and metabolic activity of hDFs and hMSCs in the sprayed Fib and FibD hydrogels. A) Representative confocal images at days 1, 7, 14, and 21, with live cells stained in green (calcein AM) and dead cells stained in red (EthD‐I); scale bars: 250 µm. B) Cell viability (%), at days 1, 7, 14, and 21 (*n* = 3 per condition and day), analyzed using one‐way ANOVA with Tukey's post‐hoc test, Welch's ANOVA with Tamhane's T2 post‐hoc test, or Kruskal–Wallis with Dunn's post‐hoc test, as appropriate. C) Cell metabolic activity, measured as fluorescence intensity (AU), at days 0, 1, 3, 5, 7, 14, and 21 (*n* = 3 per condition), analyzed using a two‐way ANOVA test, followed by Tukey's post‐hoc test. Statistical significance: *p < 0.05, **p < 0.01, ***p < 0.005.

Cell viability and metabolic activity of sprayed hydrogels are shown in **Figure** **4**. Confocal images are depicted in Figure 4A, with live cells in green and dead cells in red. hDFs showed cell viability rates between 84.3–96.4% in the Fib hydrogels and between 92.1–97.6% in the FibD hydrogels, whereas hMSCs had viability rates between 87.3–95.2% in the Fib hydrogels and between 91–96% in the FibD hydrogels. For both hDFs and hMSCs, viability was slightly higher in the FibD hydrogels than in the Fib hydrogels for each day, although only statistically significant for the hDFs on day 1. After 21 days, there was a mild reduction in hMSCs viability in the Fib hydrogels, but not in the FibD hydrogels (Figure 4A,B). Similar to cell viability, the metabolic activity of both hDFs and hMSCs was well maintained through 3 weeks, with maximum peaks after 3–7 days, which then started decreasing moderately and stabilized between 14 and 21 days. Nonetheless, hDFs and hMSCs metabolic activity was always higher in the FibD hydrogels than in the Fib ones during the experiment (Figure 4C). Concerning the control hydrogels (Figure S3, Supporting Information), both hDFs and hMSCs also showed higher metabolic activity and viability in the FibD hydrogels than in the Fib ones in all cases. Moreover, when comparing sprayed hydrogels to the pipetted controls (Figure S4, Supporting Information), the latter showed higher viability and metabolic activity than sprayed hydrogels for both hDFs and hMSCs, although differences were not statistically significant for cell viability. Despite this, sprayed samples still maintained good levels of cell viability and metabolic activity, which progressively neared those of the pipetted controls over the days, and FibD samples presented less significant differences between sprayed and pipetted hydrogels than Fib ones. The number of live and dead cells counted for each condition of the viability assay is detailed in Table S1 (Supporting Information).

#### 3.3.2 Scratch Wound Healing Assay

**Figure 5:**
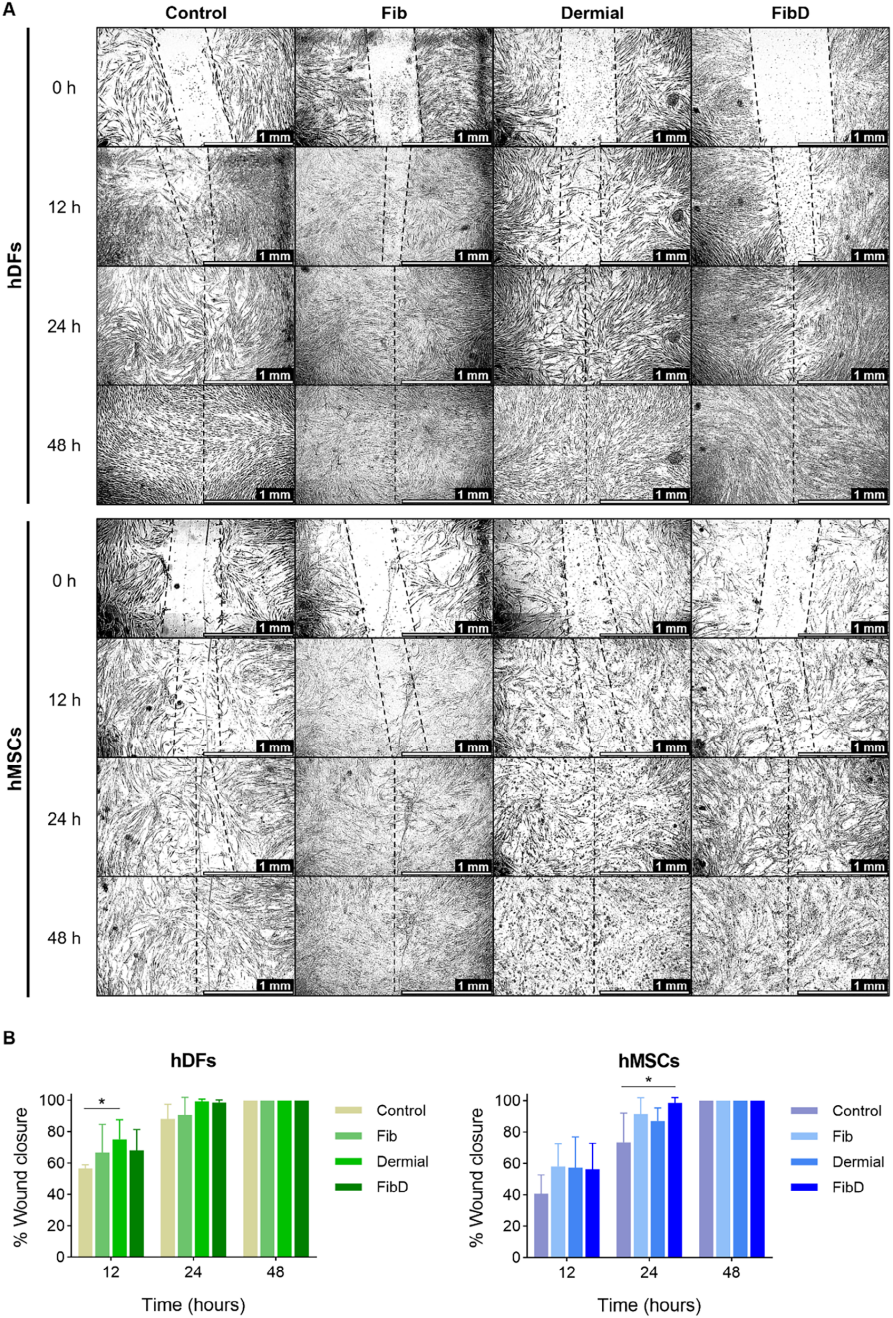
Scratch wound healing assay of hDFs and hMSCs treated with normal culture media (Control) or culture media supplemented with Fib, Dermial, or FibD (*n* = 6 per condition). A) Representative microscopy images; scale bars: 1 mm. B) % of wound closure for hDFs and hMSCs after 12, 24, and 48 h. Statistical analysis was performed using a mixed‐effects model with the Geisser–Greenhouse correction, followed by Dunnett's post‐hoc test. Statistical significance: *p < 0.05.

A scratch assay was carried out to assess the potential of the biomaterials used for the fibrinogen‐based bioinks to promote wound healing by analyzing 2D cell migration. **Figure** **5**A shows representative microscopy images of wounds over time, and the % of wound closure, measured as the reduction of the wound areas compared to the starting point (0 h), is represented in Figure 5B. For both hDFs and hMSCs, wounds treated with culture media supplemented with Fib, Dermial, and FibD showed a higher wound closure percentage than controls (wounds treated with normal culture medium) after 12 and 24 h, although only statistically significant for hDFs between control and Dermial at 12 h, and for hMSCs between control and FibD at 24 h. Furthermore, hDF wounds treated with Dermial and with FibD, as well as hMSC wounds treated with FibD, reached a wound closure close to 100% after 24 h, displaying a faster wound healing rate than the control group. All wounds were closed after 48 h.

### 3.4 In Vivo Wound Healing Assay

#### 3.4.1 Wound Healing and Clinical Evaluation

**Figure 6:**
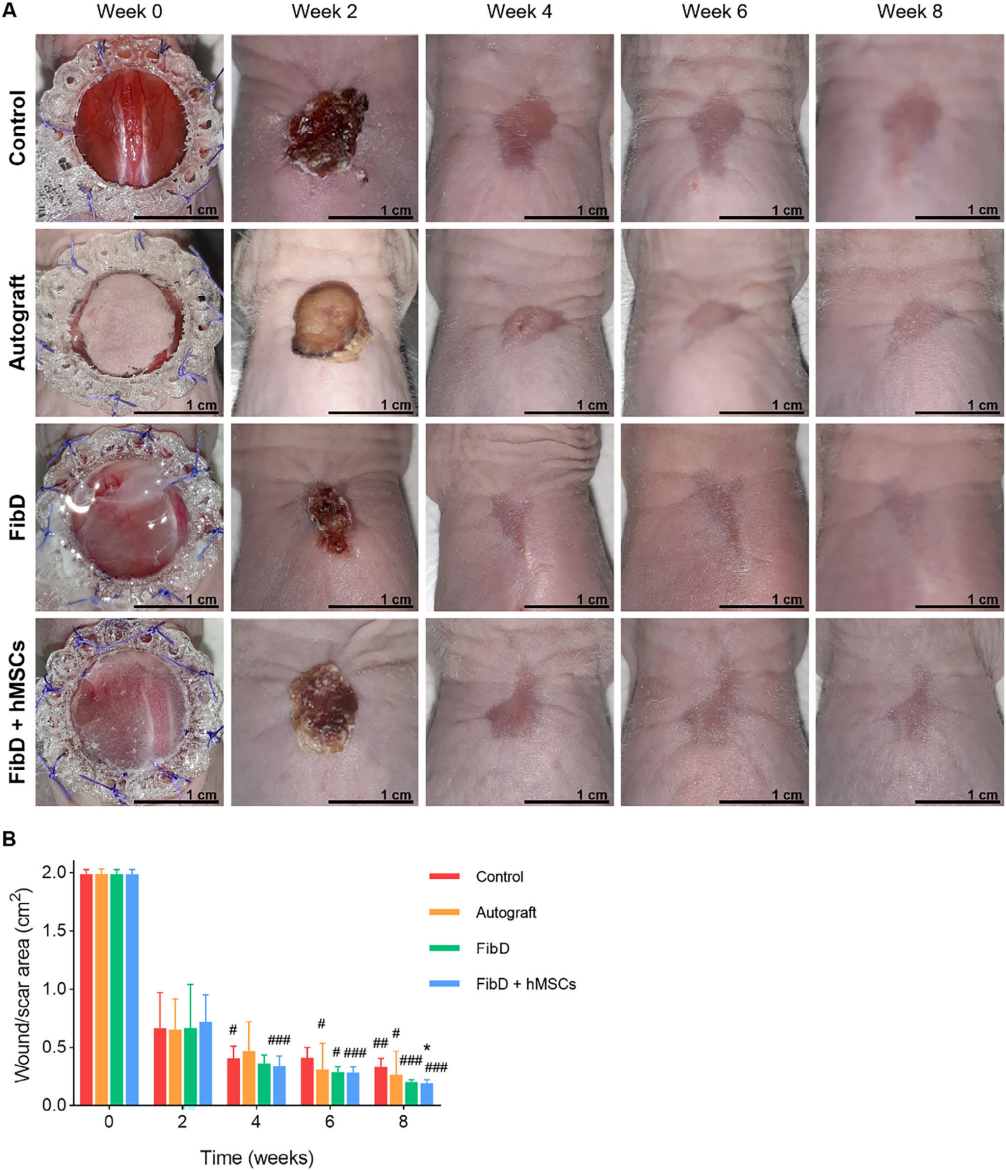
A) Macroscopic images of wound healing process over time (weeks 0, 2, 4, 6, and 8) for non‐treated mice (Control) or mice treated with Autograft, FibD, or FibD + hMSCs; scale bars: 1 cm. B) Quantitative evaluation of wound/scar area through time for all groups (*n* at week 0, 2, 4, 6, and 8 = 8, 8, 8, 4, and 4, respectively). Statistical analysis was performed using an ANOVA test followed by Dunnett's post‐hoc test, or Kruskal–Wallis followed by Dunn's post‐hoc test, as appropriate. Statistical significance compared to Control: *p < 0.05. Statistical significance compared to week 2: #*p* < 0.05; ##*p* < 0.01; ###*p* < 0.005.

The surgical procedure was well tolerated by all animals without complications. All groups of mice achieved appropriate wound stabilization after 4 weeks. For the cellular spray condition, hMSCs were chosen as they can be used as an allogeneic treatment, given their immunomodulatory properties.^[^ ^56^ ^]^ Mice treated with Autograft, FibD, and FibD + hMSCs presented a slightly faster wound repair than Control mice. After 8 weeks, skin regeneration was more effective for the FibD and FibD + hMSCs groups, while the Control group had a worse wound repair (**Figure** **6**A). Visual evaluation of scars was further assessed using an adaptation of the POSAS scale (**Table** **1**), where the total score revealed that mice treated with FibD and with FibD + hMSCs presented comparable results to mice treated with Autograft, while the Control group showed the worst results.

**Table 1:** POSAS scale results after 8 weeks.

	Vascularity^a)^	Pigentation^a)^	Thickness^a)^	Relief^a)^	Pliability^a)^	Surface area^a)^	Total score^b)^
Control	4	4	3	2	1	4	18
Autograft	3	2	2	2	1	3	13
FibD	3	2	3	2	1	3	14
FibD + hMSCs	3	2	3	2	1	2	13

^a)^ All items are scored on a scale ranging from 1 (“like normal skin”) to 10 (“worst imaginable scar”);

^b)^ The lowest score, 6, reflects normal skin, whereas the highest score, 60, reflects the worst imaginable scar.

Results by visual clinical evaluation (Figure 6A) were consistent with the quantitative analysis of the wound/scar area (Figure 6B), where the FibD and FibD + hMSCs groups presented a faster progression in wound closure. Significant differences were observed in the scar area between the FibD + hMSCs group and the Control group at 8 weeks. Additionally, scar area reduction from week 2 to week 8 was most pronounced in the FibD + hMSCs group, which showed significant differences at weeks 4, 6, and 8 compared to week 2. The FibD group also demonstrated notable improvements, with significant differences at weeks 6 and 8, indicating a superior wound closure compared to the Control and Autograft groups. While the Autograft treatment resulted in moderate improvements compared to the Control, the latter consistently exhibited the poorest wound healing outcomes. These results suggest that the FibD + hMSCs treatment was the most effective in accelerating wound healing, followed by the FibD treatment.

#### 3.4.2 Homeostasis Analysis

**Figure 7:**
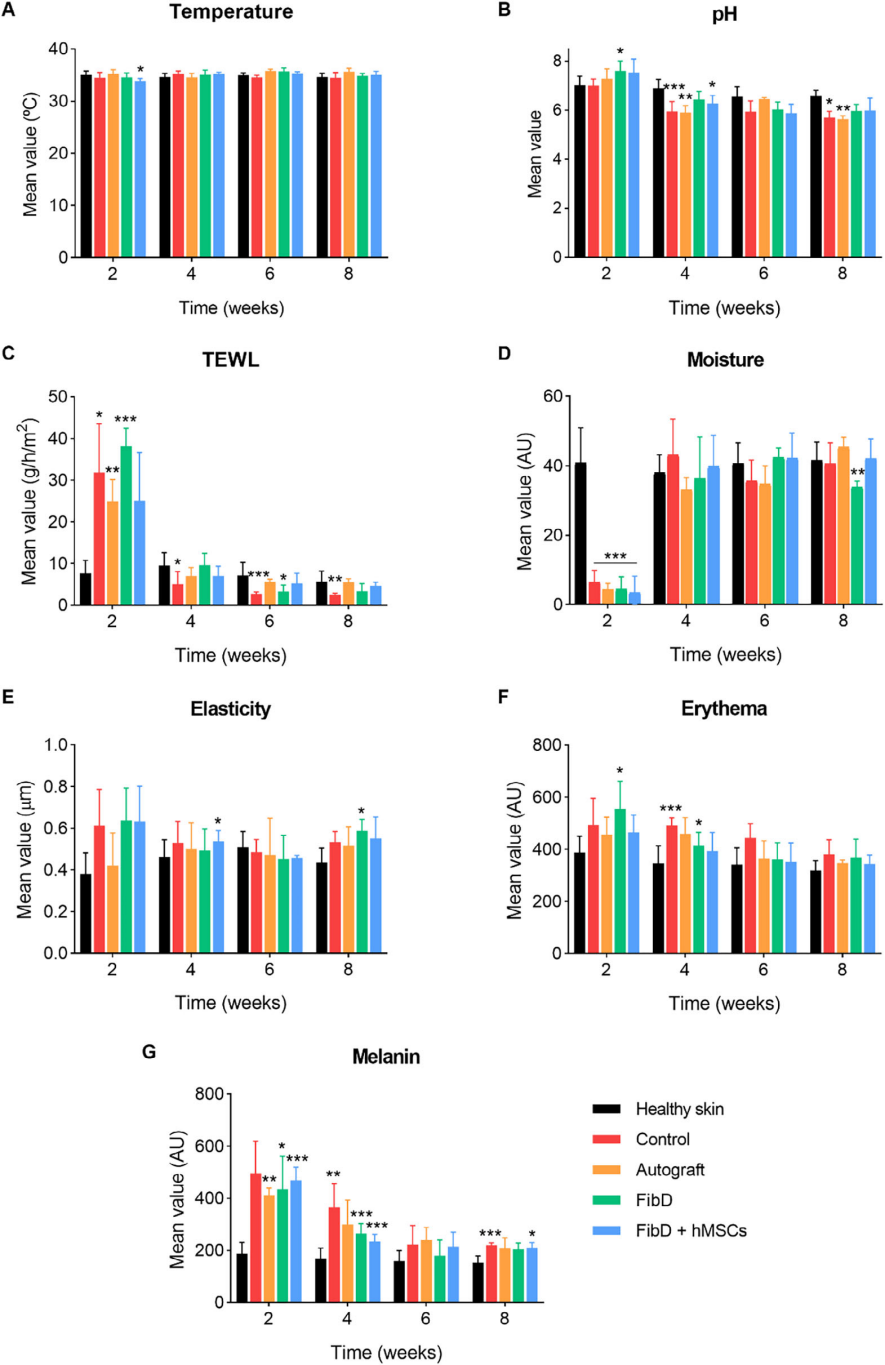
Analysis of homeostasis per parameter: A) Temperature; B) pH; C) TEWL; D) Moisture; E) Elasticity; F) Erythema; and G) Melanin. Graphics show the regenerated wounds/scars results for each treatment group (Control, Autograft, FibD, and FibD + hMSCs) against the mean values of healthy skin of all groups. Results per week were calculated as the mean value of all mice measured at each time of study: Control, Autograft, FibD, and FibD + hMSCs (*n* at week 2, 4, 6, and 8 = 8, 8, 4, and 4, respectively); Healthy skin (*n* at week 2, 4, 6, and 8 = 32, 32, 16, and 16, respectively). Statistical analysis was performed using a mixed‐effects model with the Geisser–Greenhouse correction, followed by Tukey's post‐hoc test. Statistical significance compared to healthy skin: *p < 0.05, **p < 0.01, ***p < 0.005.

Several homeostasis parameters were monitored through 8 weeks, comparing the regenerated wounds or scars to areas of healthy skin for each group of mice (Control, Autograft, FibD, or FibD + hMSCs) (**Figure** **7**; Figure S5, Supporting Information). For all groups, temperature values (Figure 7A) remained similar to those of healthy skin during the experiment, with no significant differences found after 8 weeks. On the contrary, the pH of wounds/scars decreased below healthy skin values after 4 weeks for all groups, but with the FibD and FibD + hMSCs groups having no significant differences between scars and healthy skin after 8 weeks (Figure 7B). At week 2, Autograft and FibD + hMSCs were the groups with TEWL levels closer to healthy skin values; but then TEWL decreased significantly after 2 weeks, reaching healthy skin levels for all groups except the Control group, which showed significant differences compared to healthy skin (Figure 7C). For all groups, moisture values of wounds/scars were significantly much lower than those of healthy skin at week 2, but they recovered to healthy skin values after 4 weeks, with only the FibD group showing lower values than healthy skin at week 8 (Figure 7D). Regarding elasticity (Figure 7E), Autograft was the group that the most similar values compared to healthy skin through the 8 weeks, while Control, FibD, and FibD + hMSCs groups had higher values at week 2 (although not significant), and FibD also at week 8. Finally, erythema (Figure 7F) and melanin (Figure 7G) showed a similar tendency for all groups, starting with wound/scar values being significantly higher than healthy skin and decreasing through the weeks until achieving levels close to healthy skin after 6–8 weeks.

#### 3.4.3 Histology and Immunofluorescence

**Figure 8:**
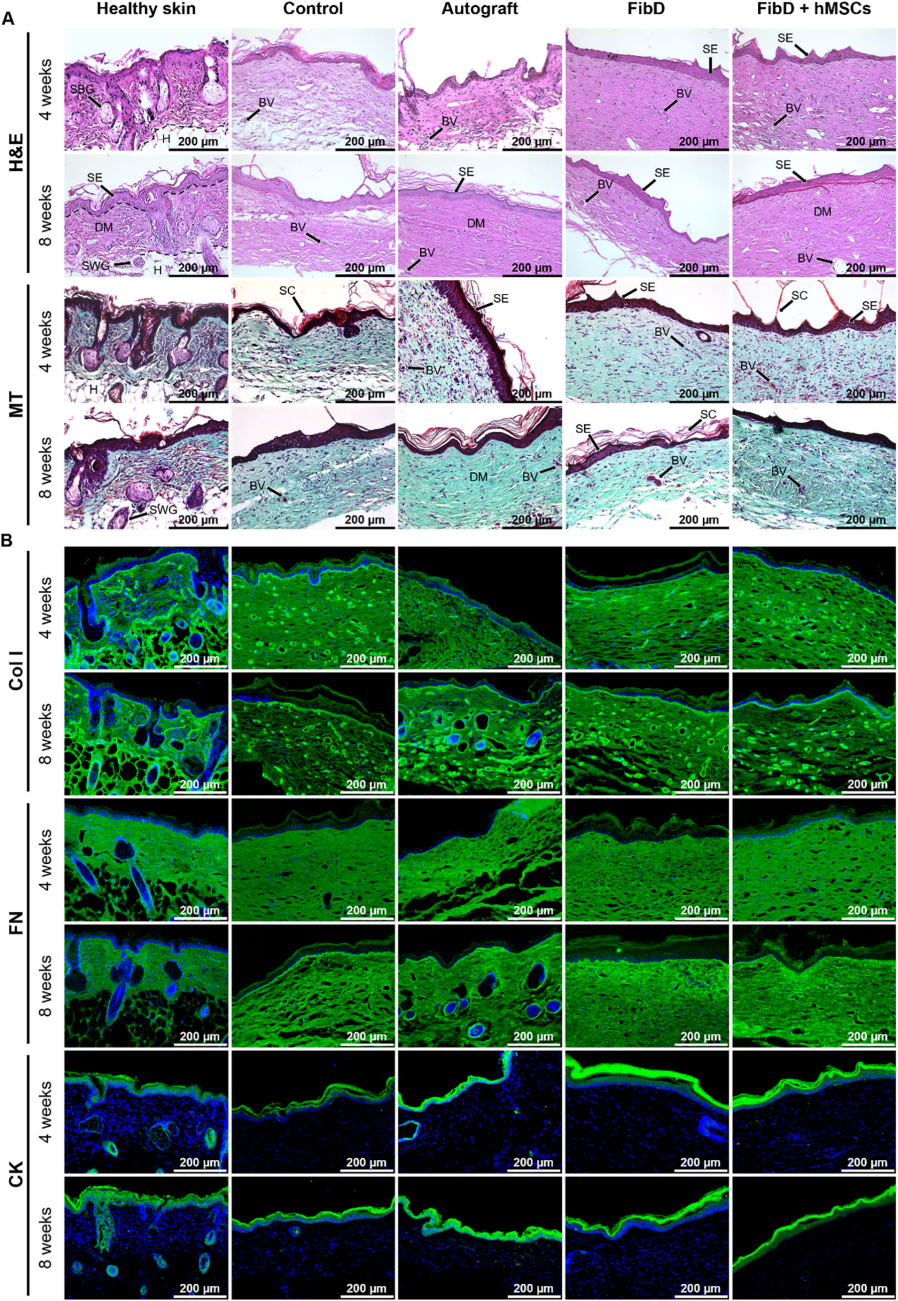
A) H&E and MT histological staining of native and regenerated skin samples of mice after 4 and 8 weeks. In H&E‐stained sections, nuclei appear purple, the epidermis stains in shades of dark pink to red due to keratin content, and the collagen‐rich dermis appears in lighter pink tones. In MT staining, collagen fibers are stained blue‐green, nuclei appear dark purple to black, and cytoplasm and keratinized epidermal layers appear dark red. Black arrows signaling: BV (blood vessels), DM (dermal matrix), H (hypodermis), SBG (sebaceous gland), SC (stratum corneum), SE (stratified epidermis), SWG (sweat gland). B) Col I, FN, and CK immunofluorescence staining of native and regenerated skin samples of mice after 4 and 8 weeks. Col I and FN stain the dermis, while CK stains the epidermis (all in green). Nuclei are counterstained with DAPI (blue). Scale bars: 200 µm.

The histological staining with H&E and MT depicted an adequate regeneration of the dermis and epidermis 4 and 8 weeks after the surgical procedure for all groups (**Figure** **8**A). After 4 weeks of healing, the treated conditions (Autograft, FibD, and FibD + hMSCs groups) showed a denser and more compact dermal matrix, evidenced by the presence of collagen‐rich areas stained light pink in H&E and blue‐green in MT, whereas the Control group's dermis appeared looser and less organized, and was not as dense until 8 weeks. At this later time point, dermal remodeling was evident in all groups, with increased matrix density and dermal thickness. Epidermal regeneration was observed in all samples by 4 weeks, although the formation of a stratified epidermis was more evident in the treated groups. The stratum corneum, recognizable by its dark pink/red staining (H&E/MT), was frequently visible, although partially detached or fragmented, likely due to the histological sectioning. Additionally, small blood vessels were observed in most samples, being more clearly defined in the FibD and FibD + hMSCs groups. However, skin appendages like sebaceous and sweat glands, clearly observable in native skin, were not observed in the regenerated skin samples, at least during the evaluation period.

In accordance with the histological results, the immunofluorescence staining (Figure 8B) showed strong and widespread expression of Col I and FN in all groups after 4 and 8 weeks, demonstrating a good regeneration of the dermis. The regeneration of the epidermis in all groups was evidenced by the expression of CK, revealing the presence of a continuous epithelial layer by 4 weeks. CK expression was more pronounced and organized in treated conditions compared to the Control group.

These results suggest that the Autograft, FibD, and FibD + hMSCs treatments are capable of achieving a regenerated dermis and epidermis most similar to native healthy skin after just 4 weeks, with the Control (no treatment) needing more time to obtain similar results.

## 4 Discussion

The biofabrication of skin substitutes has gained special attention in recent years as an alternative to overcome the limitations of conventional skin autografting. Nevertheless, the manufacturing of some substitutes may be difficult, expensive, and time‐consuming, sometimes needing several weeks to be available. Early treatment of the wound is essential to achieve a better functional outcome, reduce scar formation, and avoid infections and related complications.^[^ ^8^, ^57^ ^]^ The use of skin sprays offers a potential tool for wound healing applications, as they allow the delivery of different biomaterials and/or cell suspensions to the wound bed, being able to treat extensive wounds and even areas with unfavorable topography, rapidly and with great ease.^[^ ^13^, ^58^ ^]^ Patients can be treated immediately in an outpatient setting, reducing their stay at the hospital, minimizing complications, and allowing them to restore their normal mobility much faster.^[^ ^59^, ^60^, ^61^ ^]^

Despite the advantages of skin cell sprays, their translation to clinical practice is still scarce.^[^ ^13^ ^]^ To tackle this problem, it is necessary to develop more advanced regenerative spray products with biomolecules that provide an ideal microenvironment and signaling for the survival and motility of the cells to accelerate the entire wound healing process. In addition, it is also important to improve the configuration of the devices to enable the loading and spraying of diverse bioink types while preserving their integrity. To date, the vast majority of skin spray studies use syringes with spray pumps or regular airbrushes, which can only spray one material at a time. Only a few previous studies have also developed pneumatic‐assisted cell delivery devices, but these are still limited to a single syringe for delivering material.^[^ ^60^, ^62^, ^63^ ^]^ Furthermore, the few spraying devices that allow the delivery of two materials (generally fibrinogen and thrombin solutions) are double syringes attached to spray tips.^[^ ^13^ ^]^ In this study, we have developed the first dual‐head airbrush‐based device designed for the treatment of skin lesions, with a double spraying system that allows the delivery of two different materials either simultaneously or successively.

Given its essential role in wound healing and its hemostatic properties, fibrinogen has been the most widely used biomaterial for skin sprays.^[^ ^14^ ^]^ Our bioink was formulated with fibrinogen and supplemented with a GAGs/Col‐based matrix containing a high concentration of HA, dermatan sulfate, chondroitin sulfate, and Col.^[^ ^46^, ^50^ ^]^ In addition to the advantages of fibrinogen, HA promotes tissue regeneration during the wound healing process, reduces scar formation, maintains skin moisture, and has immunomodulatory properties.^[^ ^2^, ^64^ ^]^ Moreover, the GAGs/Col‐based matrix used in this study has been reported to possess regenerative, anti‐aging, and antioxidant properties in vitro, and stimulate the synthesis of Col, elastin, and GAGs, which are essential to provide mechanical strength to the regenerated skin.^[^ ^46^, ^50^ ^]^ Previous studies have reported the use of fibrinogen‐based bioinks for skin treatments,^[^ ^26^, ^65^, ^66^, ^67^, ^68^, ^69^ ^]^ however, to the best of our knowledge, this is the first study showing a bioink based on a fibrinogen/GAGs/Col combination for application in skin sprays. While existing clinical spray products focus mostly on fibrin sealants (e.g., TISSEEL, VISTASEAL) or simple cell suspensions (e.g., ReCell), the use of biomimetic ECM‐based components such as our GAGs/Col‐supplemented bioink, alone or in combination with cells, is not yet represented in commercially available systems. Moreover, our dual‐head device enables this type of co‐delivery, expanding the therapeutic potential of spray‐based treatments.

A frequent drawback of many skin spray studies is the little relevance they give to the spraying parameters, as the use of different parameters can influence results significantly, especially in cell sprays.^[^ ^13^ ^]^ Depending on the duration, cells are generally damaged by hydrostatic, shear, and elongation stresses while being sprayed when passing through the nozzle,^[^ ^70^ ^]^ but they are also damaged when they impact the receiving surface. Cell viability has been reported to be negatively affected by a smaller nozzle diameter, higher spraying pressure and velocity, higher viscosity of the transporting fluid, and stiffness of the receiving surface. On the contrary, a larger cell‐containing droplet diameter and a longer spraying distance are favorable for cell viability.^[^ ^3^, ^71^, ^72^ ^]^ Although the viability of epidermal cells after spraying has been demonstrated in several studies,^[^ ^21^, ^66^, ^72^ ^]^ cells can be damaged without having their membrane disrupted, so the post‐aerosolization proliferative capacity must be assessed as well as the viability.^[^ ^70^ ^]^ We optimized the spraying pressure of our system to 15 psi, as higher pressures resulted in decreased metabolic activity and proliferative capacity of the cells, in agreement with Harkin et al., who reported that the viability and mitochondrial enzyme activity of keratinocytes immediately after aerosolization were reduced with spraying pressures above 20 psi.^[^ ^66^ ^]^ This is presumably because cells under larger pressures are also subjected to more demanding shear stresses at the nozzle. In our setup, each nozzle produced a narrow spray cone (≈12–16°), and droplet size analysis showed most droplets were below 100 µm, providing a well‐defined spray pattern. The consistency of droplet sizes and distribution across replicates indicated a controlled and reproducible atomization process at the selected parameters, well‐suited for uniform bioink deposition.

Once the spraying parameters were determined, the physicochemical properties of the inks and hydrogels were characterized. The Fib and FibD inks presented acidic pH values of 6.12 ± 0.19 and 6.1 ± 0.17, respectively, adequate for their application in skin wounds.^[^ ^73^, ^74^, ^75^, ^76^ ^]^ The swelling or liquid absorption ability of hydrogels influences the entrance of fluids and the transport of nutrients into the hydrophilic hydrogel matrices, as well as cell migration and proliferation.^[^ ^42^, ^77^ ^]^ The Fib and FibD hydrogels presented a significant swelling capacity, reaching their maximum level after only two days. The FibD hydrogels showed a relatively higher swelling ratio in comparison to the Fib hydrogels, anticipated due to the high water retention capability of HA and the higher porosity of the matrix when HA is assembled into the fibrin matrix.^[^ ^36^, ^44^ ^]^ This behavior was expected since higher swelling ratios of fibrin‐HA hydrogels compared to fibrin have been reported previously.^[^ ^78^ ^]^ On the other hand, the biodegradability of the hydrogels is important as it can interfere with cell growth and tissue regeneration.^[^ ^79^ ^]^ The Fib hydrogels degraded faster than FibD hydrogels, probably due to the stabilization of the fibrin matrix and the regulation of its degradation by HA.^[^ ^44^ ^]^ Fib hydrogels degraded after 17 weeks, while it took 20 weeks for FibD hydrogels to degrade completely. Hydrogels serving as skin substitutes should degrade in an appropriate period to allow the vascularization and the deposition of ECM molecules for the formation of the new granulation tissue, but previously, the hydrogel should be present for enough time as it serves as a platform for cell migration, adhesion, and proliferation.^[^ ^44^, ^80^ ^]^ Therefore, the prolonged degradation of the FibD hydrogels could be beneficial for a better regeneration process of the skin tissue. Moreover, this slower degradation profile may be particularly advantageous in chronic wounds, where ECM remodeling is often dysregulated and tissue regeneration is significantly delayed, requiring a hydrogel matrix that remains present and bioactive for extended periods.^[^ ^81^, ^82^ ^]^

In biofabrication, a high viscosity of the bioink or biomaterial ink is essential to maintain the 3D structure of the resulting construct, and specifically in skin sprays, to prevent runoff of the sprayed material and extend its contact time with the wound bed. However, as mentioned previously, a higher viscosity of the transporting fluid affects cell viability negatively.^[^ ^28^, ^71^ ^]^ Fibrinogen inks have been reported to exhibit a Newtonian fluid behavior,^[^ ^83^ ^]^ which is in agreement with our results, where our Fib ink maintained a constant viscosity of 1.163 ± 0.095 mPa s. On the other hand, the supplementation with the GAGs/Col‐based matrix resulted in a shear‐thinning behavior of the FibD ink. The shear‐thinning characteristics of a fibrinogen/HA bioink have been previously reported,^[^ ^78^ ^]^ as a favorable behavior for the use of bioinks in biofabrication techniques such as skin sprays or 3D bioprinting, given that the decrease in viscosity under high shear stress (during dispensing/extrusion) reduces cell damage, and the subsequent recovery of high viscosity under low shear stress (after dispensing/extrusion) helps to prevent leakage of the material and maintain the 3D shape.^[^ ^78^, ^84^ ^]^

Fibrin is one of the softest natural fibers, with a high elastic deformation capacity and large stretchability, but its mechanical properties can be optimized by adjusting the precursor concentrations of fibrinogen and thrombin.^[^ ^29^, ^31^ ^]^ Cell morphology, migration, and protein expression of many cell types, including fibroblasts, are affected by the stiffness of their substrate. In fibrin matrices, the stiffness increases with higher concentrations of fibrinogen and thrombin.^[^ ^85^, ^86^, ^87^ ^]^ In our study, we used fibrinogen and thrombin concentrations of 10 mg mL^−1^ and 50 U mL^−1^, respectively, following the manufacturer's recommendations. The Young's moduli of our hydrogels ranged between 0.169 ± 0.025 and 0.312 ± 0.041 kPa, falling within the reported 0.001 to 10 kPa range of substrate stiffness necessary for cell attachment.^[^ ^87^ ^]^ In acellular hydrogels, a slight reduction in stiffness and an increase in viscoelasticity were observed in FibD hydrogels compared to Fib hydrogels. The reduction of fibrin stiffness when mixed with non‐crosslinked HA has been previously reported.^[^ ^78^ ^]^ Moreover, the spraying process did not affect the stiffness and viscoelasticity of hydrogels significantly, in comparison to the pipetted controls. For all cellular hydrogels, the Young's Modulus was higher after 21 days in culture compared to day 1, while the storage and loss moduli were lower at 21 days. This could be explained by the formation of ECM by cells within the fibrin matrix, and by the slight shrinkage of the hydrogels observed as time progressed, due to cell proliferation,^[^ ^87^ ^]^ making hydrogels stiffer and less viscoelastic over time.

Thereafter, the biocompatibility of the bioink was analyzed in vitro with hDFs and hMSCs, two of the most commonly studied cells in tissue‐engineered skin substitutes.^[^ ^13^, ^88^ ^]^ Sprayed Fib and FibD hydrogels exhibited good metabolic activity with high viability levels through the 3 weeks for both hDFs and hMSCs, with the FibD hydrogels yielding relatively better results in all cases, with cells seemingly more numerous and elongated than in the Fib hydrogels, demonstrating the beneficial effect of the addition of the GAGs/Col‐based matrix on cells.^[^ ^46^, ^50^ ^]^ However, there was a mild deceleration in metabolic activity after approximately one week in most cases, at the same time as a slight contraction of the hydrogels was observed. It has been shown that this shrinkage reduces the porosity of the fibrin matrices, resulting in a more rigid matrix that restrains cell spreading and matrix remodeling. The reduction of the pore size also decreases the permeability of nutrients, and could subsequently lead to cell starvation in the inner core of the hydrogels. Similarly to stiffness, porosity also depends on the fibrinogen and thrombin concentrations, as they affect fibers' thickness and density.^[^ ^87^, ^89^, ^90^ ^]^ Nonetheless, the concentrations of fibrinogen and thrombin in our formulation were optimized to allow nutrient diffusion, and despite the slight shrinkage of gels, the viability and metabolic activity still showed a good tendency after 3 weeks. Similarly, it has been reported that although fibrinogen mixed with non‐crosslinked HA resulted in reduced porosity, the viability of hMSCs was not negatively affected thanks to the stimulation of cell attachment by HA.^[^ ^29^, ^78^ ^]^ On the other hand, despite sprayed hydrogels showing lower viability and metabolic activity than control hydrogels (which was expected due to the stresses suffered by the cells during the spraying process), sprayed samples still maintained acceptable levels of cell viability and metabolic activity, which recovered to similar levels than those of controls over time, revealing that this bioink could be suitable for its application with this spray system. Moreover, the supplementation of the fibrinogen‐based hydrogels with the GAGs/Col matrix presented better results than fibrinogen alone in all cases, indicating the positive effect of these biomaterials. Concerning the scratch wound healing assay, the supplementation of the culture medium with Fib, Dermial, or a combination of both (FibD) resulted in a faster closure of the scratch wound for both hDFs and hMSCs, in comparison to the non‐treated controls. These results demonstrate that fibrinogen and the GAGs/Col‐based matrix can promote the acceleration of the migration and proliferation of hDFs and hMSCs, which is in agreement with the model proposed by Weigel et al. that indicates a specific binding interaction between fibrin, HA, and other cross‐linking components as responsible for the stimulation of cell migration into the wound and the subsequent skin tissue reconstruction processes.^[^ ^44^ ^]^ In line with this, Col and HA are known to play a key role in wound healing by supporting cell adhesion, migration, and ECM remodeling.^[^ ^91^ ^]^

In summary, compared to fibrinogen alone (Fib), the FibD formulation consistently demonstrated superior performance across multiple parameters, including higher swelling capacity, slower degradation rate, shear‐thinning behavior, enhanced cell viability and metabolic activity, and greater wound closure in scratch assays.

In this work, we also studied the healing process of full‐thickness skin wounds in a murine model, evaluating the closure rates, homeostasis parameters, and histological structure of regenerated wounds treated with autografts or with our sprayed acellular and cell‐loaded bioink, in comparison to non‐treated control wounds. After the in vitro results, we decided to use the (acellular) FibD ink and the FibD + hMSCs bioink as our spray treatments for the in vivo assay, given the physicochemical and biological advantages and beneficial effects conferred by the GAGs/Col‐based matrix, as well as its previously reported regenerative properties.^[^ ^46^, ^50^ ^]^ Despite the FibD + hDFs bioink exhibiting promising wound healing properties, hMSCs were selected as a preferred cell source for the in vivo assay due to their immunomodulatory properties which encourage their use as an allogeneic cellular therapy.^[^ ^56^ ^]^ In addition, hMSCs present numerous advantages for the enhancement of tissue regeneration, such as the secretion of paracrine factors, the promotion of angiogenesis, or their ability to regulate the function of other cell types involved in the wound healing process, helping to attenuate scar formation while reducing the inflammatory response.^[^ ^56^ ^]^ An important difference between human and murine skin is their healing mechanisms, as the former heals through re‐epithelialization, while the latter heals through contraction.^[^ ^92^ ^]^ Therefore, for the in vivo model we used a customized 3D printed b‐TPUe splint in order to prevent wound contraction during the first 2 weeks, thus ensuring that the observed wound closure was due to the different applied treatments and not wound contraction. Our results revealed that, while non‐treated control mice wounds showed the worst results, mice with the FibD and FibD + hMSCs spray conditions presented a faster wound closure and that their wound repair was comparable to the ones treated with Autografts, the *gold standard* in clinical practice.

Moreover, all groups of mice achieved an adequate recovery of the homeostasis parameters after 8 weeks. However, the Autograft, FibD, and FibD + hMSCs groups yielded the best results, while the Control group had the biggest differences compared to healthy skin. The epidermal barrier plays a crucial role in thermoregulation.^[^ ^93^ ^]^ The temperature of regenerating wounds remained similar to that of healthy skin through the 8 weeks for all groups, indicating the correct restoration of this homeostatic function. The disruption of the skin barrier leads to an increase in pH,^[^ ^74^ ^]^ which was observed when comparing wounds to healthy skin after 2 weeks. As wounds healed over time, the pH decreased, and after 8 weeks, the difference in pH between wounds/scars and healthy skin was more statistically significant in Control and Autograft mice than in mice treated with FibD, and FibD + hMSCs. As well, when the integrity of the skin barrier is damaged, there is a higher TEWL, and consequently, a reduction of moisture.^[^ ^94^ ^]^ This was observed at week 2, where wounds presented TEWL levels much higher and moisture levels much lower than healthy skin; but after 4 weeks, wounds reached levels similar to healthy skin for both parameters in all groups, although the Control group had the biggest differences. However, the FibD + hMSCs group was the one with the TEWL values with the smallest difference compared to healthy skin at week 2, and values almost identical to healthy skin from week 4 to 8. Given that in the Autograft group the wounds were covered with a biopsy of healthy skin, the mechanical properties, concretely the elasticity, of the regenerated wounds/scars of the Autograft group were the most similar to healthy skin during all the experiment, while the rest of the groups presented higher values than healthy skin at week 2. After a skin injury, erythema (i.e., the redness of the skin) is caused by hyperemia in superficial capillaries.^[^ ^95^ ^]^ The erythema levels of the wounds of the Autograft and FibD + hMSCs groups were the most similar to healthy skin during the experiment, while the rest of the groups progressively decreased until approaching healthy skin levels, with Control showing the highest values from week 4 to 8. Pigmentation is a result of the melanin produced by melanocytes, which appear at the wound site once it has re‐epithelialized. Therefore, hypopigmentation would be expected during the first weeks of the healing process. However, it is not atypical to observe hyperpigmentation of the wounds as they heal, caused by the activation of melanocytes by the inflammatory response during wound healing.^[^ ^96^ ^]^ This was observed especially in the case of the Control group, which had the highest melanin levels during most of the experiment, whereas the Autograft, FibD, and FibD + hMSCs groups had slightly lower levels, more similar to healthy skin, which could be explained due to the anti‐inflammatory properties of the bioink.

Finally, the histological and immunofluorescence staining revealed that, after just 4 weeks, the Autograft, FibD, and FibD + hMSCs groups presented a dense dermal matrix that expressed Col I (the major structural protein of the reticular dermis) and FN (the dominant glycoprotein in the provisional dermal matrix assembled for the formation of granulation tissue, with a crucial role in wound healing).^[^ ^97^, ^98^ ^]^ Moreover, the expression of CK, which is a family of proteins that have been demonstrated to be markers of epidermal differentiation,^[^ ^99^, ^100^ ^]^ revealed a good regeneration of the epidermis. On the other hand, non‐treated Control wounds required a longer time to achieve similar results.

Altogether, these results demonstrate that even though all groups of mice were able to restore their skin barrier and functions correctly after 8 weeks, the spray treatment with the acellular or cell‐loaded FibD bioink offered faster and better functional and aesthetic outcomes than non‐treated mice. Although these results were comparable to those of the Autograft, the spray treatment with the cellular and acellular FibD bioink presents the advantage of avoiding the need to create a secondary wound to obtain an autologous biopsy. In addition, this bioink would allow to have an allogeneic treatment that could be applied in situ instead of requiring weeks for its availability. Future in vivo studies could extend the evaluation period to 12–24 weeks to allow the investigation of the long‐term regenerative capacity, the durability of the treatment, and scar formation over time.

Furthermore, given the positive results obtained with FibD even without cells, this ink could certainly be translated to clinical practice as a medical device, with less stringent requirements than the regulatory pathway associated with cellular skin sprays. For this aim, although the current version of the spraying device is a functional prototype, it incorporates key features that could facilitate future clinical translation, such as the use of disposable syringes, the possibility of autoclaving metallic nozzles, and modular elements that allow for thorough cleaning and sterilization. For clinical use, the design could be further adapted using materials approved for medical applications and following standardized sterilization protocols to meet regulatory requirements. This could open more possibilities for the use of this ink and the spraying device for different applications, such as hemostasis, surgical sealing, chronic wounds, other skin pathologies, or even cosmetic treatments. Overall, these findings support the clinical therapeutic potential of the bioink in combination with the spraying device as a promising tool for the immediate treatment of skin wounds. However, this study has certain limitations, including the use of a murine model, which may not fully replicate the complexity of human wound healing. Future investigations should incorporate large animal models to improve the translational relevance of the results and evaluate long‐term outcomes such as immune response, tissue integration, and scar formation.

## 5 Conclusion

We have developed the first dual‐head spraying device based on an airbrush system with application in the treatment of cutaneous wounds. We have also formulated a new fibrinogen‐based bioink supplemented with a GAGs/Col‐based matrix ingredient of high HA concentration, which also contains dermatan sulfate, chondroitin sulfate, and Col. These biomimetic biomaterials, characteristic of the native skin's ECM, provide beneficial properties for skin wound healing. Our novel FibD bioink proved to be suitable for its spray application through our device, forming hydrogels with suitable physicochemical, mechanical, and biological properties for their use as skin substitutes. The hydrogels exhibited high and stable cellular viability and acceptable cell metabolic activity, and demonstrated the promotion of faster wound healing and tissue regeneration in vivo, obtaining comparable results to those of autografts, while avoiding the downsides of the latter. The FibD bioink has the potential to be used as an autologous treatment, using dermal cells such as hDFs obtained from the patient; as an allogeneic treatment, using allogeneic hMSCs that could help to prevent immune response or the need to obtain an autologous biopsy; or even as an acellular treatment, which could be more easily translated to clinical practice. Furthermore, this spray device enables the use of a variety of bioinks with different compositions, as long as they have adequate rheological properties. As a matter of fact, the syringes of the new spray device fabricated in this study can be quickly and easily replaced by others loaded with diverse bioinks, which could be useful to spray alternate layers of different compositions. In addition, when used in combination with adequate bioinks, this device could be used for other different clinical applications, like hemostasis, other skin pathologies, or cosmetic treatments. Overall, this combination of our bioink together with the spraying device could offer a promising therapeutic tool for the immediate treatment of skin wound patients.

## Conflict of Interest

P.G.‐M. is employed by Bioiberica S.A.U. The rest of the authors declare no conflict of interest.

## Author Contributions

P.P.‐B. dealt with conceptualization, methodology, investigation, acquisition, analysis, interpretation of data, writing the original draft, review and editing, and visualization. D.N.‐G. dealt with the design and creation of the spraying device. C.C.‐W. dealt with assistance with in vitro and in vivo experiments and writing the review and editing. J.d.V. dealt with assistance with rheological experiments and data interpretation. P.G.‐M. dealt with the provision of resources and writing the review and editing. J.M.E. dealt with assistance with animal experimentation. E.L.‐R. and J.A.M. dealt with conceptualization, writing the review and editing, supervision, project management, and funding acquisition. All authors have read and approved the final manuscript.

## Supporting information



Supporting Information

## Data Availability

The data that support the findings of this study are available on request from the corresponding author. The data are not publicly available due to privacy or ethical restrictions.
