# Supplementary material for: A Novel Sprayable Fibrinogen/Glycosaminoglycans/Collagen‐Based Bioink for Skin Wound Healing Applied by a Handheld Dual‐Head Airbrush
Source: Adv Healthc Mater. 2025 Jul 25;15(5):e00702. doi: 10.1002/adhm.202500702 (PMC12864579; doi:10.1002/adhm.202500702)
Supplement: Supplementary file 1 — Supporting Information [file ADHM-15-0-s001.docx]

A Novel Sprayable Fibrinogen/Glycosaminoglycans/Collagen-Based Bioink for Skin Wound Healing Applied by a Handheld Dual-Head Airbrush

**Paula Pleguezuelos-Beltrán^1,2,3,4,5^, Daniel Nieto-García^4,5,6,7^, Carlos Chocarro-Wrona^1,2,3,4,5^, Juan de Vicente^5,8^, Patricia Gálvez-Martín^9^, José Manuel Entrena^10,11^, Elena López-Ruiz^1,2,4,5,12,^*, Juan Antonio Marchal^1,2,3,4,5,^*.**

^1^ Biopathology and Regenerative Medicine Institute (IBIMER), Center for Biomedical Research (CIBM), University of Granada, 18016 Granada, Spain.

^2^ Instituto de Investigación Biosanitaria ibs.GRANADA, 18012 Granada, Spain.

^3^ Department of Human Anatomy and Embryology, Faculty of Medicine, University of Granada, 18016 Granada, Spain.

^4^ BioFab i3D Lab – Biofabrication and 3D (bio)printing Laboratory, 18016 Granada, Spain.

^5^ Excellence Research Unit "Modeling Nature" (MNat), University of Granada, Granada, Spain.

^6^ Center of Advanced Scientific Research (CICA), University of La Coruña, 15001 A Coruña, Spain.

^7^ Complex Tissue Regeneration Department, MERLN Institute for Technology-Inspired Regenerative Medicine, Maastricht University, Universiteitssingel 40, 6229 ER Maastricht, The Netherlands.

^8^ F2N2Lab, Magnetic Soft Matter Group, Department of Applied Physics, Faculty of Sciences, University of Granada, 18003 Granada, Spain.

^9^ R&D Human Health, Bioibérica S.A.U., 08950 Barcelona, Spain.

^10^ Institute of Neuroscience, Center for Biomedical Research (CIBM), University of Granada, 18016 Granada, Spain.

^11^ Animal Behavior Research Unit, Scientific Instrumentation Center, University of Granada, 18003 Granada, Spain.

^12^ Department of Health Sciences, University of Jaén, 23071 Jaén, Spain.

*Correspondence to: Elena López-Ruiz, [elruiz@ujaen.es](mailto:elruiz@ujaen.es) (E.L.R.), Juan Antonio Marchal, [jmarchal@go.ugr.es](mailto:jmarchal@go.ugr.es) (J.A.M.)

**Abstract:** In the last years, different biofabrication methods have gained special attention for the production of skin substitutes that overcome the limitations of conventional skin grafting. Skin sprays represent a promising technology for treating cutaneous wounds as they can deliver both cells and biomaterials to the wound bed in a fast and easy approach, covering extensive wound surfaces. The aim of this study is to develop a novel bioink based on fibrinogen supplemented with a glycosaminoglycans (GAGs)/collagen (Col)-based matrix, containing hyaluronic acid, dermatan sulfate, chondroitin sulfate, and Col, in combination with an innovative dual-head airbrush-based spraying device. The fibrinogen/GAGs/Col-based bioink is loaded with human mesenchymal stromal cells or human dermal fibroblasts, and its physicochemical and mechanical properties are analyzed, as well as cell viability, metabolic activity, and *in vitro* wound healing. Finally, its skin wound healing properties are studied in an *in vivo* excisional wound healing murine model. The bioink forms hydrogels with satisfactory physicochemical, mechanical, and biological properties, capable of promoting wound healing and tissue regeneration *in vivo* with outcomes comparable to those of autografts. The novel spray system and bioink show the potential to serve as a therapeutic tool for the clinical treatment of cutaneous wounds.

**Keywords:** skin; spray; fibrin; bioink; biofabrication.

**SUPPORTING INFORMATION**


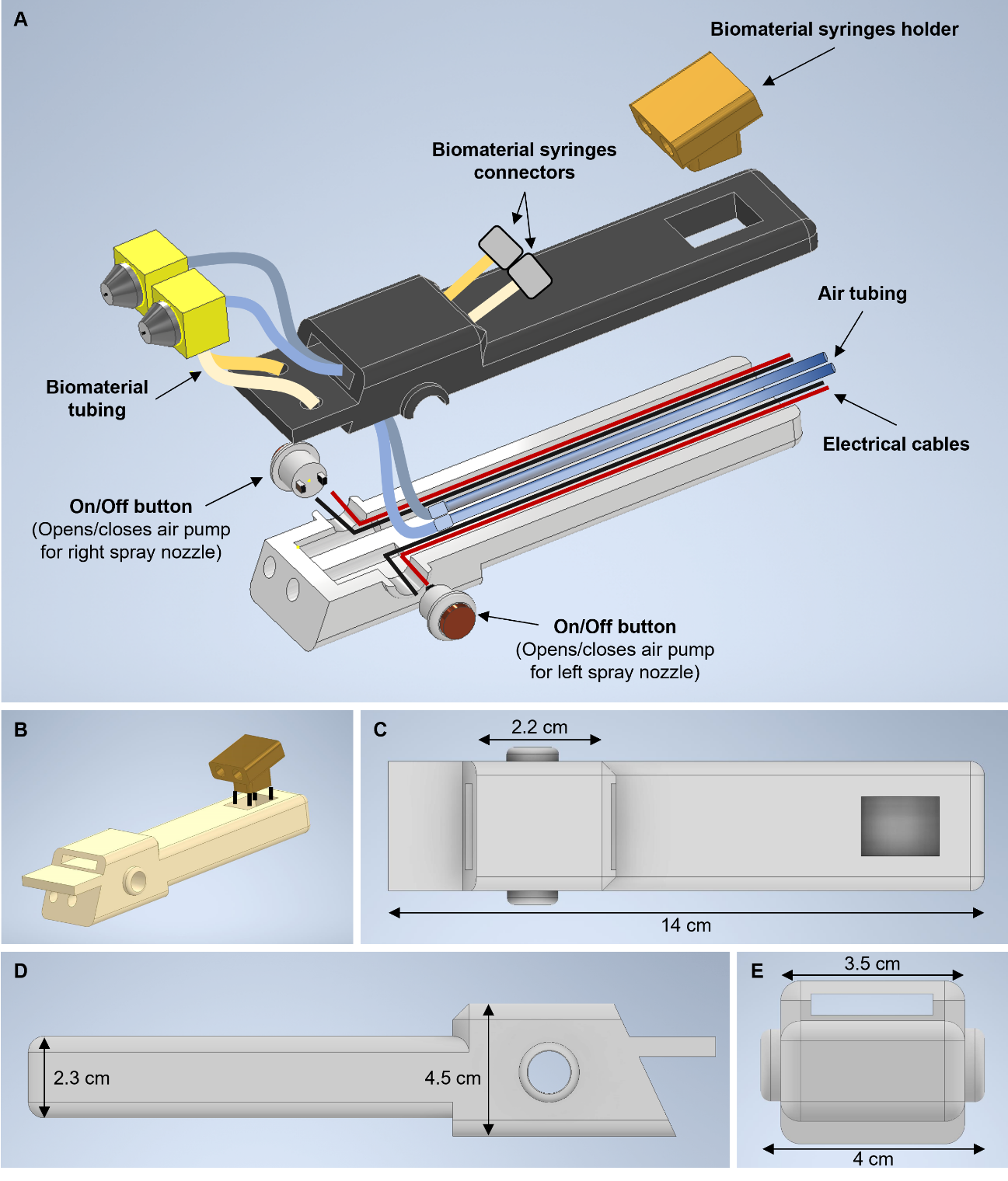


**Figure S1**. Airbrush’s 3D CAD design. (A) Schematic representation of the device. CAD design of the 3D printed chassis: (B) overview; (C) top view; (D) lateral view; and (E) back view.


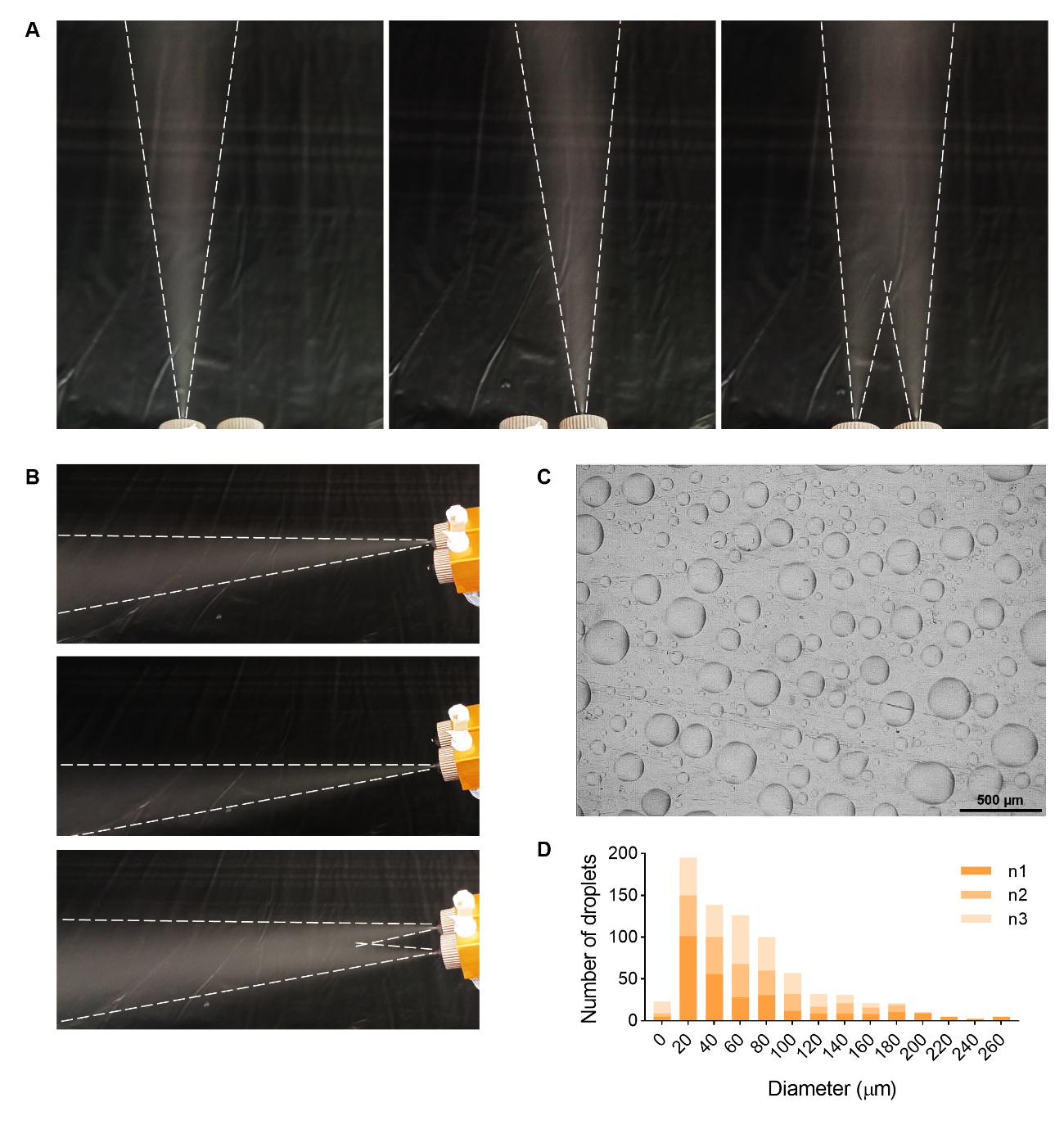


**Figure S2**. (A) Top view and (B) lateral view of the spray cone angle generated by the device, using a spraying pressure of 15 psi and at a 10 cm distance, visualized against a black background. (C) Representative image of droplet deposition pattern on a parafilm sheet; scale bar: 500 µm. (D) Histogram showing the frequency distribution of droplet diameters (total n = 768 droplets) measured from 3 independent spray depositions on parafilm (n1 = 291, n2 = 224, and n3 = 253 droplets). Most droplets (75.91%) were below 100 µm. Total mean droplet diameter = 64.98 ± 51.13 µm.


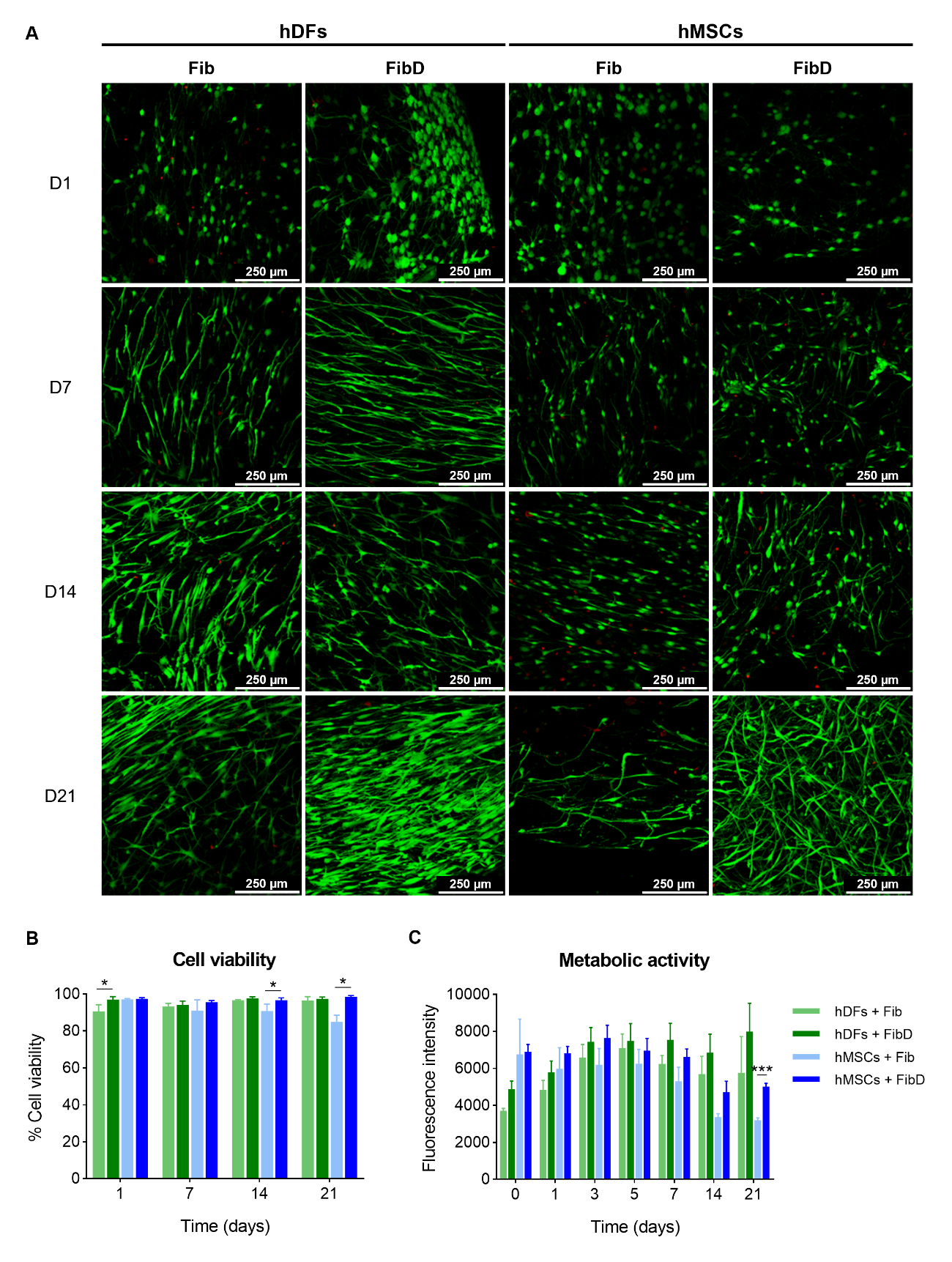


**Figure S3**. Cell viability and metabolic activity of hDFs and hMSCs in the pipetted (Control) Fib and FibD hydrogels. (A) Representative confocal images at days 1, 7, 14, and 21, with live cells stained in green (calcein AM) and dead cells stained in red (EthD-I); scale bars: 250 µm. (B) Cell viability (%), at days 1, 7, 14, and 21 (n = 3 per condition and day), analyzed using one-way ANOVA with Tukey’s post-hoc test, Welch’s ANOVA with Tamhane's T2 post-hoc test, or Kruskal-Wallis with Dunn’s post-hoc test, as appropriate. (C) Cell metabolic activity, measured as fluorescence intensity (AU), at days 0, 1, 3, 5, 7, 14, and 21 (n = 3 per condition), analyzed using a two-way ANOVA test, followed by Tukey’s post-hoc test. Statistical significance: *p < 0.05, ***p < 0.005.


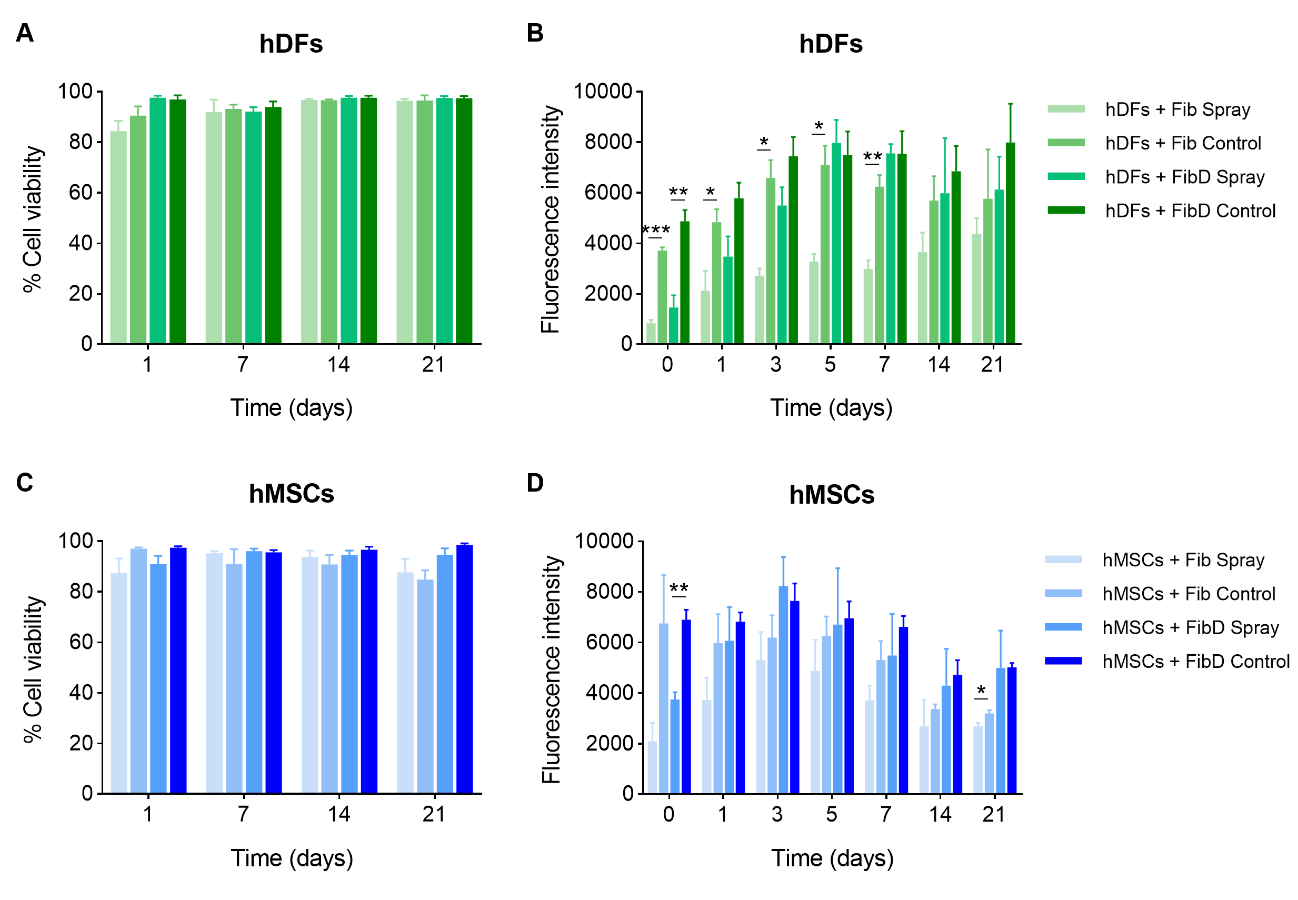


**Figure S4**. (A) Cell viability (%) of hDFs in the pipetted (Control) and sprayed Fib and FibD hydrogels, at days 1, 7, 14, and 21 (n = 3 per condition). (B) Cell metabolic activity, measured as fluorescence intensity (AU), of hDFs in the pipetted (Control) and sprayed Fib and FibD hydrogels, at days 0, 1, 3, 5, 7, 14, and 21 (n = 3 per condition). (C) Cell viability (%) of hMSCs in the pipetted (Control) and sprayed Fib and FibD hydrogels, at days 1, 7, 14, and 21 (n = 3 per condition). (D) Cell metabolic activity, measured as fluorescence intensity (AU), of hMSCs in the pipetted (Control) and sprayed Fib and FibD hydrogels, at days 0, 1, 3, 5, 7, 14, and 21 (n = 3 per condition). Statistical analysis for cell viability (A, C) was conducted using one-way ANOVA with Tukey’s post-hoc test, Welch’s ANOVA with Tamhane’s T2 post-hoc test, or Kruskal-Wallis with Dunn’s post-hoc test, as appropriate. For cell metabolic activity (B, D), two-way ANOVA followed by Tukey’s post-hoc test was applied. Statistical significance: *p < 0.05, **p < 0.01, ***p < 0.005.

**Table S1**. Quantification of live and dead cells in the viability assay.

|  |  |  | **n1** | | **n2** | | **n3** | |
| --- | --- | --- | --- | --- | --- | --- | --- | --- |
|  |  | **Day** | **Live** | **Dead** | **Live** | **Dead** | **Live** | **Dead** |
| **Spray** | **hDFs + Fib** | 1 | 304 | 78 | 332 | 47 | 309 | 51 |
|  |  | 7 | 385 | 27 | 412 | 33 | 329 | 38 |
|  |  | 14 | 402 | 15 | 415 | 14 | 406 | 12 |
|  |  | 21 | 410 | 14 | 468 | 23 | 453 | 14 |
|  | **hDFs + FibD** | 1 | 447 | 13 | 494 | 17 | 436 | 6 |
|  |  | 7 | 483 | 56 | 466 | 36 | 590 | 40 |
|  |  | 14 | 545 | 8 | 402 | 11 | 494 | 13 |
|  |  | 21 | 621 | 24 | 632 | 9 | 555 | 11 |
|  | **hMSCs + Fib** | 1 | 317 | 67 | 360 | 51 | 289 | 23 |
|  |  | 7 | 414 | 18 | 372 | 16 | 305 | 19 |
|  |  | 14 | 488 | 22 | 421 | 21 | 440 | 48 |
|  |  | 21 | 504 | 96 | 476 | 44 | 492 | 58 |
|  | **hMSCs + FibD** | 1 | 217 | 24 | 235 | 29 | 209 | 14 |
|  |  | 7 | 248 | 11 | 271 | 9 | 316 | 17 |
|  |  | 14 | 374 | 20 | 309 | 12 | 332 | 29 |
|  |  | 21 | 424 | 24 | 422 | 28 | 430 | 20 |
| **Control** | **hDFs + Fib** | 1 | 306 | 53 | 290 | 22 | 239 | 17 |
|  |  | 7 | 446 | 36 | 448 | 22 | 420 | 40 |
|  |  | 14 | 522 | 20 | 482 | 20 | 467 | 14 |
|  |  | 21 | 574 | 12 | 533 | 18 | 543 | 31 |
|  | **hDFs + FibD** | 1 | 256 | 11 | 358 | 3 | 192 | 9 |
|  |  | 7 | 381 | 14 | 366 | 27 | 279 | 25 |
|  |  | 14 | 415 | 6 | 403 | 16 | 430 | 8 |
|  |  | 21 | 533 | 19 | 511 | 15 | 537 | 6 |
|  | **hMSCs + Fib** | 1 | 230 | 8 | 245 | 6 | 216 | 7 |
|  |  | 7 | 316 | 75 | 356 | 22 | 375 | 21 |
|  |  | 14 | 399 | 41 | 466 | 68 | 429 | 24 |
|  |  | 21 | 456 | 98 | 519 | 75 | 508 | 93 |
|  | **hMSCs + FibD** | 1 | 233 | 5 | 224 | 8 | 235 | 5 |
|  |  | 7 | 318 | 18 | 322 | 12 | 331 | 13 |
|  |  | 14 | 452 | 24 | 420 | 9 | 438 | 13 |
|  |  | 21 | 624 | 4 | 508 | 10 | 512 | 13 |

Total number of live and dead cells quantified using ImageJ (Fiji) for each condition (n = 3) at days 1, 7, 14, and 21, in both sprayed and control (pipetted) samples.


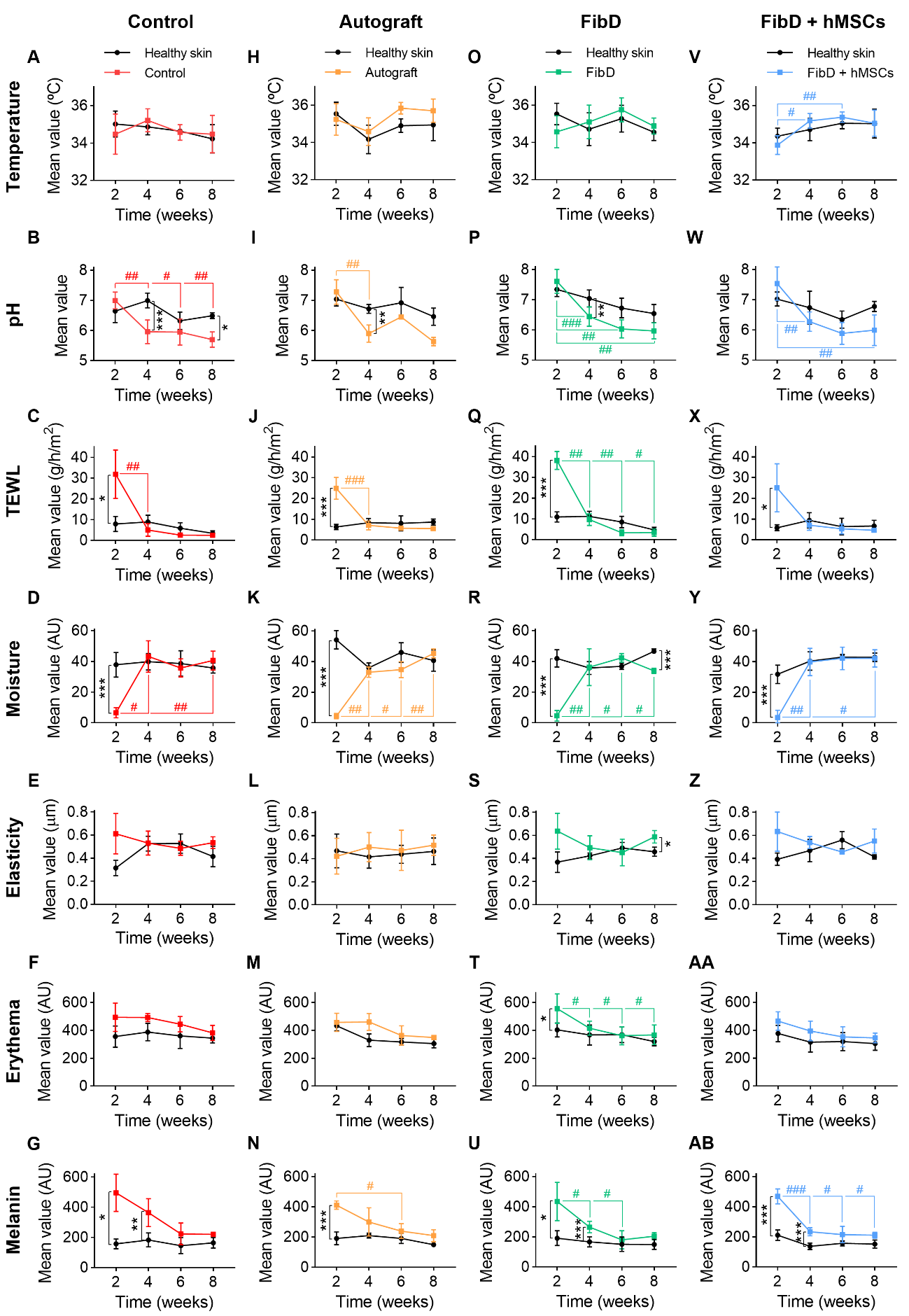


**Figure S5**. Analysis of homeostasis parameters per week and group. Graphics show results of regenerated wounds/scars against healthy skin for each treatment group: Control (A–G), Autograft (H–N), FibD (O–U), and FibD + hMSCs (V–AB). Results per week were calculated as the mean value of all mice measured at each time of study (n at week 2, 4, 6, 8 = 8, 8, 4, 4). Statistical analysis was performed using a mixed-effects model with the Geisser-Greenhouse correction, followed by Sidak’s post-hoc test for comparison to healthy skin, and Dunnett’s post-hoc test for comparison to week 2. Statistical significance compared to healthy skin: *p < 0.05; **p < 0.01; ***p < 0.005. Statistical significance compared to week 2: #p < 0.05; ##p < 0.01; ###p < 0.005.
